# Prioritizing disease-associated missense variants with chemoproteomic-detected amino acids

**DOI:** 10.1016/j.ajhg.2025.04.017

**Published:** 2025-05-23

**Authors:** Maria F. Palafox, Lisa Boatner, Blake R. Wilde, Heather Christofk, Keriann M. Backus, Valerie A. Arboleda

**Affiliations:** 1 Department of Human Genetics, David Geffen School of Medicine, UCLA, Los Angeles, CA 90095, USA; 2 Department of Pathology and Lab Medicine, David Geffen School of Medicine, UCLA, Los Angeles, CA 90095, USA; 3 Department of Biological Chemistry, David Geffen School of Medicine, UCLA, Los Angeles, CA 90095, USA; 4 Department of Chemistry and Biochemistry, College of Arts and Sciences, UCLA, Los Angeles, CA 90095, USA; 5 Molecular Biology Institute, UCLA, Los Angeles, CA 90095, USA; 6 DOE Institute for Genomics and Proteomics, UCLA, Los Angeles, CA 90095, USA; 7 Jonsson Comprehensive Cancer Center, UCLA, Los Angeles, CA 90095, USA; 8 Eli and Edythe Broad Center of Regenerative Medicine and Stem Cell Research, UCLA, Los Angeles, CA 90095, USA; 9 Department of Computational Medicine, David Geffen School of Medicine, UCLA, Los Angeles, CA 90095, USA

**Keywords:** genomics, proteomics, chemoproteomics, missense variation, cysteine, clinical genetics, Mendelian disease, cancer predisposition, tyrosine, lysine

## Abstract

Missense variants are the most common type of protein-altering genetic variation. Due to their wide-ranging potential functional consequences, missense variants are challenging to interpret and, as a result, are often classified as unknown pathogenicity or as variants of uncertain significance (VUSs). Genomic-based predictive tools have made significant inroads into the challenge of accurately pinpointing functional missense variants by providing genome-wide assessments of deleteriousness or potential pathogenicity. Complementary to these tools, here we provide an initial study into the utility of harnessing protein-based measures of amino acid reactivity to delineate functionally significant missense variants. These reactivity measurements, which are generated using mass spectrometry-based chemoproteomic methods, have already proved capable of pinpointing functional sites on proteins, which provide the added value of delineating potential sites suitable for drug-development efforts. Here, using published chemoproteomic datasets for three specific privileged amino acids, cysteine, lysine, and tyrosine, we assessed the utility of proteomic reactivity measurements to identify clinically important variants and regions within monogenic-disease-associated genes. We found that genes where amino acids are detected via chemoproteomics are enriched for monogenic-disease phenotypes, indicative of functional importance. Chemoproteomic-detected amino acids (CpDAAs) are enriched at and around sites with known pathogenic missense variants when assessed with either one- or three-dimensional protein structures. To further illustrate the utility of our findings, we found that regions at or around CpDAAs in fumarate hydratase (FH) were enriched for VUSs and pathogenic variants, which we validate through demonstration of an altered FH oligomerization state. Collectively, our study highlights the potential of chemoproteomic and genetic data integration for enhancing the identification of functional genetic variants and protein sites with potential value for drug-development efforts.

## Graphical abstract

**Figure:**
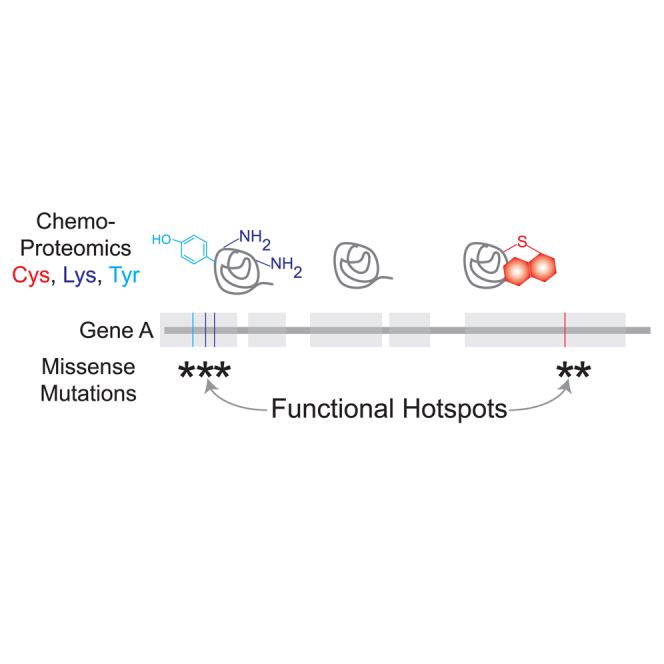

## Teaser

Mass spectrometry-based chemoproteomics can provide a high-throughput assessment of the likely functionality of specific sites on proteins. Here, we show that chemoproteomic measures of protein-based amino acid reactivity effectively highlight clinically important variants in disease-associated genes. These findings should have relevance in enhancing variant interpretation and drug development.

## Introduction

Genetic diversity gives rise to both biological diversity and disease. Across all protein-coding genetic variants, missense variants are particularly challenging to functionally define. Less than 2% of missense variants have been classified based on American College of Medical Genetics guidelines.^1^ Further exemplifying this challenge, in the ClinVar database,^2^ over half of all missense variants are annotated as variants of uncertain significance (VUSs). Likely many of these variants are functionally significant, as indicated by 38% of Mendelian disorders caused by missense variation.^2^ The annotation of missense variants associated with clinical phenotypes has far outpaced our understanding of critical protein domains and how mutations disrupt specific functions. Even for genes currently associated with a disease phenotype, establishing the causal role of specific missense variants remains challenging, and many individuals with suspected rare genetic diseases are left without a definitive diagnosis.^3^

Missense variants are difficult to functionally annotate for several reasons. Unlike nonsense and frameshift mutations, which almost invariably result in a truncated protein,^4^ the impact of missense variants can have a wide range of functional consequences, including no effect, enhanced or decreased protein activity, altered stability, mistargeted subcellular localization, and disrupted post-translational regulation. The functional impact of a missense change can be partly rationalized by the Grantham distance^5^ (a measure of the chemical differences between two amino acids), the functional importance of the substituted residue (e.g., known catalytic residue or site of post-translational modification), and the structural context of the amino acid residue (e.g., buried or solvent accessible). Notably, missense variants that are buried in the structure and away from solvent accessible sites are more likely to lead to protein misfolding.^6^^,^^7^ Further illustrating the complexities of accurately assigning function to missense variants, domain-specific missense variants often lead to different clinical phenotypes compared to other variants in the same gene.^8^

Toward addressing this gap, many methods have emerged to guide the discovery of functional missense variants, including, most notably, unsupervised natural language computational models that can predict deleteriousness or potential pathogenicity.^9^^,^^10^^,^^11^^,^^12^ One widely used example of these variant effect predictors (VEPs) is the combined annotation-dependent depletion (CADD) score,^13^ which provides an indication of deleteriousness when mutated.^14^^,^^15^ While VEPs have proved highly useful, they still lack precision in accurately assigning the pathogenicity of missense variants.^16^

Recent studies, including our own,^14^^,^^15^^,^^17^^,^^18^ revealed that mass spectrometry-based chemoproteomics, a method that uses chemical probes to pinpoint likely functional sites on proteins, can complement genetic data to guide identification of functional hotspots in proteins on a proteome-wide scale. For example, in our recent work, we revealed that cysteine residues with high chemoproteomic measures of intrinsic nucleophilicity (a proxy for functionality) are correlated with high CADD scores. Recent work by Cravatt and colleagues combined base editing with chemoproteomic measures of potential druggability, which revealed that a subset of potentially druggable cysteine residues are essential and represent targetable vulnerabilities in cancer.^19^ Further demonstrating the clinical relevance of these findings, cysteine-reactive molecules have emerged as blockbuster drugs for a number of different indications, spanning cancers (e.g., afatinib, sotorasib,^20^^,^^21^ ibrutinib) and autoimmune disorders (e.g., dimethyl fumarate [DMF], ibrutinib). Our recent work in cancer datasets has further expanded the chemoproteomics sites and covalent probes that can precision-target missense variants across a wide range of human cancers.^22^

Here, we pursue a multi-omic approach to define and delineate opportunities for large-scale chemoproteomic data in guiding the identification of likely functional and therapeutically relevant missense variants. Through analysis of published chemoproteomics datasets for cysteine-, lysine-, and tyrosine-reactive probes, we first find that proteins identified via chemoproteomics are enriched for monogenic-disease-associated genes—these data provided initial evidence that chemoproteomic detection is indicative of functional significance. Further delineation at the domain, sequence, and 3D-structure levels revealed that chemoproteomic-detected amino acids (CpDAAs) are enriched proximal to missense variants, particularly for pathogenic variants. Deployment of these findings on the case-study protein fumarate hydratase (FH), associated with renal cell cancer, corroborated the marked enrichment of CpDAAs proximal to both pathogenic and VUS, both in linear sequence and 3D space. In sum, by integrating proteome-wide chemoproteomic measures with missense variants in known disease-causing genes, we demonstrate the added value that chemoproteomics detection brings to identifying functional missense variants.

## Methods

### Curation and standardization of chemoproteomics datasets

In selecting protein-profiling datasets to combine into a single quality-controlled inventory, attention was paid to variables such as choice of cell line(s) profiled, broad-spectrum probes used, and concentrations and incubation times of probes tested. The highly reactive chemical probes IA-alkyne, STP-alkyne, and HHS-465 were previously used to profile the intrinsic reactivity of cysteine, lysine, and tyrosine residues, respectively. The chemoproteomics data from published manuscripts are available at https://github.com/ArboledaLab/CKY. We filtered out peptides containing multiple modified amino acids (e.g., MAC^∗^ALRC^∗^Y) and excluded peptides outside our established length parameters of 6–45 amino acids, consistent with our and other laboratory pipelines. All chemoproteomics data used in our study were confirmed to pass a second quality control step, which required peptides and residues to appear at least twice across all experimental replicates. For reactivity-profiling experiments, reactivity ratios (R_10:1_) for each residue were averaged across peptide replicates to assign a final R10:1 value. Reactivity levels were then categorized as low (R_10:1_ > 5), medium (2 < R_10:1_ ≤ 5), or high (R_10:1_ ≤ 2). After filtering and standardizing reactivity labels, we verified CpD-residue identities and positions against UniProtKB canonical peptide sequences from August 2021. A small fraction of peptides did not map to this reference version of UniProt (<1%). For a more detailed explanation of the mapping pipeline at the gene/protein level, see supplemental methods. As discussed in Palafox MF et al.,^14^ we used Ensembl v92 gene and transcript sequences as our cross-reference to UniProt because the gnomAD v2.1.1 (released in 2019) used Ensembl v92 annotations to generate its constraint scores (including pLI, LOEUF, and others) for GRCh37/hg19.^23^

### CpD gene annotation by FDA-approved drug targets and OMIM monogenic disorder genes

We used a Venn diagram approach to compare and visualize the overlap among gene sets. Using the R package VennDiagram, we represented the intersection and unique elements of the datasets. Before creating the diagrams, we preprocessed the data using the HUGO Gene Nomenclature Committee database to remove duplicates and standardize gene nomenclature, ensuring uniform comparison criteria.^14^ The US Food and Drug Administration (FDA)-approved drug-target gene set came from the Human Proteome Atlas.^24^^,^^25^ We mapped these drug targets to CpD data using the Ensembl v92 gene ID column, with CpD data standardized to UniProt canonical protein sequences compatible with this Ensembl version (*n* = 751 FDA genes; Table S3). We obtained monogenic disorders and their associated genes from the Online Mendelian Inheritance in Man (OMIM)^26^ and filtered them to include only genes associated with at least one single-gene disorder (*n* = 3,974; Table S3). While 1,243 genes overlapped between the OMIM and CpD gene lists, we further required each gene to have at least one pathogenic and one common/benign missense variant. This resulted in 926 shared genes, which we refer to as OMIM&CpD.

### Variant classification

We defined pathogenic missense variants by curating a high-confidence set of missense variants from ClinVar,^2^ a database of disease variants reported by clinical testing. High-confidence missense variants were subdivided to create a mutually exclusive set of pathogenic, benign, and VUS missense variant sets. The “background” (non-pathogenic) variants were curated through combination of gnomAD^23^ missense variants (variant allele frequency >0.05) and ClinVar variants classified as benign in these same OMIM monogenic genes. We use the background set of variants instead of simply the common/benign variant from ClinVar due to the limited counts of available common/benign missense that might bias the interpretation of our analysis.

### 1D calculations

To investigate the spatial relationships between pathogenic, common/benign, rare, and VUS missense variants to specific amino acid residue positions in proteins, we used a 1D distance analysis approach. This method calculates the absolute number of amino acids separating a missense variant position from a reference residue position, such as cysteine (Cys, C), lysine (Lys, K), or tyrosine (Tyr, Y), collectively referred to as CysLysTyr residues. These distances were assigned to unique identifiers representing the each variant-residue pair (missense variant position and CysLysTyr residue position). Distance distributions were also calculated for unique missense positions relative to CysLysTyr positions, including cases where the distance was zero, reflecting direct overlap. For each reference CysLysTyr position, a sequence window centered on the residue was analyzed to summarize the total number of unique missense variants within a defined range (e.g., ±6 amino acids). Several window sizes were tested in our analysis of non-direct spatial relationships between CysLysTyr and missense positions. Analyzing protein sequences using sliding windows of approximately six amino acids has proved effective across multiple applications. This window size is well supported by research showing that six-residue patches contain crucial protein information^27^ important for protein folding and aggregation and can effectively distinguish between amyloid and non-amyloid peptides.^28^ Although optimal window sizes may vary by prediction task,^29^ the six-residue window has become a standard choice. We tested both shorter (±3 amino acids) and longer (±15 amino acids) and saw no significant differences in properties.

Finally, only sequences with at least one pathogenic and one common/benign missense variant were used for 1D window analysis. To calculate the nearest missense variant to a given CysLysTyr, direct overlapping missense variants were excluded from the analysis. This exclusion criterion was established to mitigate potential biases in statistical comparisons between detected and undetected residue types, emphasizing proximity relationships without confounding effects from direct overlaps.

The 1D distances were analyzed to compare the enrichment of missense variants in detected versus undetected CysLysTyr residue positions or 1D-windows. Fisher’s exact test was applied to assess enrichment at CysLysTyr positions and within 1D windows, with odds ratios (ORs) calculated to determine the likelihood of variant mapping to detected CysLysTyr residue positions or windows. ORs greater than 1 indicated enrichment of pathogenic variants, while values less than 1 indicated enrichment of common/benign variants relative to undetected CysLysTyr residues.

### 3D distance calculations

Proteins with CpDAAs were cross-referenced with the Protein DataBank (PDB) downloaded June 23, 2022. All biological assembly files of entries were processed. For each CpD protein associated with a PDB, the sorting intolerant from tolerant (SIFT) database (2019 release) was used to map protein sequence residue positions to PDB structure residue positions. The author determined biological unit annotations were extracted from each PDB, as well as the exact 3D coordinates of a CpDAA. Specifically, distances were calculated with respect to locations of the following side-chain atoms for each amino acid to all other atoms of neighboring amino acids within 10 Å. For cysteine, distances were measured from the sulfhydryl group (SG) sulfur atom of cysteine residues. For lysine, distances were measured from the nitrogen atom of the L-lysine amino acid side chain (NZ atom). For tyrosine, distances were measured from the oxygen atom in the phenolic hydroxyl (OH) group. The smallest distance between terminal cysteine, lysine, or tyrosine atoms and atoms of neighboring missense variant positions were stored for statistical analyses. Multiple PDB structures assigned to a given UniProt identifier used for missense environment counting. At the amino acid level, all proximal amino acids to a detected residue were assigned a distance pair identifier, composed of the protein identifier and protein position of a given residue. The distance pairs for a single environment (in the consistent direction from missense protein position to CpDAA position) were all unique.

Our choice of distance cutoffs was determined empirically, as we tested several angstrom (Å) distances (6, 8, and 10) and did not identify significant differences between them. Importantly, many studies have extensively examined the optimal distance cutoff for analyzing protein structure networks and residue-residue interactions. A cutoff of 7–8 Å has emerged as a key parameter for protein contact analysis.^30^^,^^31^ This range effectively captures the major intramolecular interactions that influence protein stability across various secondary structures.^5^ Therefore, our data were primarily focused on the 8-Å cutoff.

Our PDB mapping pipeline provided distances from the terminal atoms of cysteine, lysine, and tyrosine residues (SG, NZ, and OH atoms, respectively) to all other atoms of neighboring residues within a maximum environment boundary set for 10 Å from the reference terminal atom coordinate.^29^ To calculate 3D environment missense burden, environment boundaries of 6, 8, and 10 Å^29^ and all unique missense alleles mapping to environment based on terminal atom coordinates for cysteine, lysine, and tyrosine residues were considered.

### Residue mapping to dbNSFPv4.2a

We mapped CysLysTyr residues to CADD scores—a key metric for predicting missense variant pathogenicity—using dbNSFPv4.2a. Since dbNSFPv4.2a uses UniProt v2019_01 while our reference uses UniProt v2021, our mapping process included a thorough sequence-verification step. This step checked both the total protein length and every amino acid position to ensure compatibility between versions. Due to this rigorous verification process and our requirement for complete CADD scores at all possible non-synonymous SNVs per codon, some CpD proteins were removed from analysis. See supplemental methods for detailed description of losses in the residue-level analysis. This careful approach guaranteed high-quality missense-level annotations across all remaining CpD protein positions.

### CADD score

To support stratification of CpDAAs with higher functional potential, the CADD metric was used based on previous demonstration of its performance across diverse classes of genes. CADD scores also have the added advantage of minimal missense values for missense predictions of all possible non-synonymous changes in the human genome. Deleterious missense predictions for possible CpDAA substitutions were also tested for significant associations to environment features such as local pathogenic variants. A mean phred score for all possible non-synonymous exchanges above 25 defined deleterious CADD scores.

## Results

### Establishing a test dataset of chemoproteomic-detected genes and CpDAAs

To improve our understanding of the relationships between chemoproteomic detection and human genetic variation, we first generated a curated dataset that combines six publicly available chemoproteomics profiling experiments covering 4,535 chemoproteomic-detected (CpD) proteins.^14^^,^^32^^,^^33^^,^^34^^,^^35^^,^^36^ These datasets report measures of the relative reactivity and covalent modification by drug-like small molecules at cysteine, lysine, and tyrosine positions, collectively referred to as CpDAAs (Table S1). All raw, filtered, and annotated datasets representing all the CpDAAs in our analysis are available at https://github.com/ArboledaLab/CKY. In total, after curation, our datasets harbor 18,827 detected residue positions within 4,535 total CpD proteins, with an average of 1.63 cysteine, two lysine, and 0.52 tyrosine sites per protein (Table S2). Nearly half of all CpD proteins (44%; 1988/4535) harbor two or more types of reactive amino acids (e.g., cysteine and tyrosine residues; Figure S1). Only 14% of proteins harbor all three types of reactive amino acids (cysteine, lysine, and tyrosine).

### CpD proteins are enriched for monogenic-disease genes and FDA drug targets

**Figure 1:**
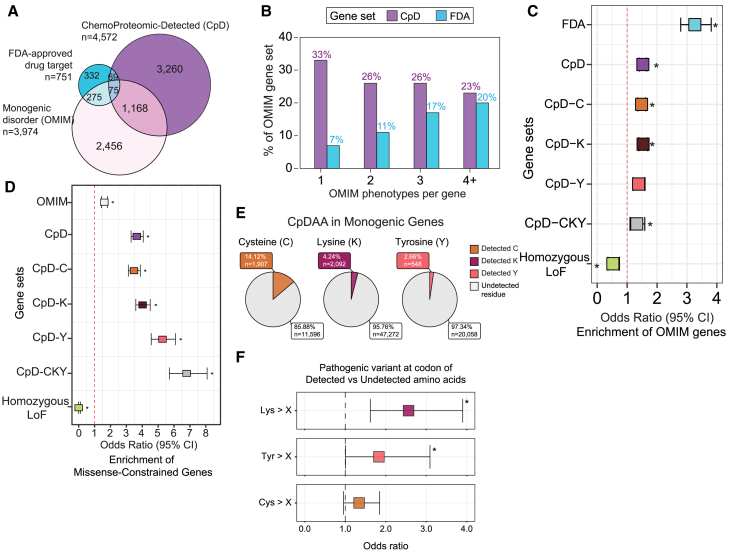
CpD proteins are enriched for pathogenic missense variants in human monogenic-disease genes (A) Venn diagram of the overlap genes between the FDA-approved drug-target gene set, gene set of chemoproteomic-detected (CpD) genes, and gene set associated with monogenic disorders (OMIM). (B) Distribution of genes from the CpD protein (purple) and FDA-approved drug target (cyan) gene sets across five levels based on the number of OMIM monogenic disorder phenotypes per gene (*x* axis). Data are based on a reference set of 20,210 total human protein-coding genes. Bar height represents the fraction of each gene set associated with a level of phenotypes counted per gene (Table S4). (C) Enrichment of OMIM genes associated with monogenic disorders across CpD and FDA-target genes. Gene set sizes: total human protein-coding genes = 16,812, OMIM = 3,703, CpD-C (Cys) = 3,239, CpD-K (Lys) = 2,488, CpD-Y(Tyr) = 1,026, CpD-CKY (CysLysTyr) = 615, FDA = 707, homozygous LoF tolerant = 285. ^∗^*p* threshold <0.007142857, all points were significant. (D) Enrichment for missense intolerant genes in gene sets related to monogenic disorders (OMIM), CpD (all proteins with any chemoproteomic-detected residue), CpD-C (proteins with CpD cysteine), CpD-Lys (CpD-lysine sites), CpD-Tyr (Cpd-tyrosine sites), and CpD-CysLysTyr (proteins with all three types of sites) and homozygous LoF-tolerant genes. We identified 1,681 genes that were depleted of missense variation, represented by the missense observed/expected upperboaund fraction (MOEUF-constrained), defined as those in Fisher’s exact test that was used to compare gene counts (Table S5). Point estimates of OR >1 indicate a gene set’s enrichment for missense-intolerant genes based on the described threshold. Significant associations are marked by asterisk (^∗^). Bonferroni correction for multiple testing. Horizontal bars show the 95% confidence interval (CI) of the OR point estimates. (E) Proportion of detected (*n* = 4,547) and undetected (*n* = 78,926) cysteine, lysine, and tyrosine residue positions in 926 OMIM&CpD proteins. (F) OR of missense categories overlapping detected versus undetected cysteine, lysine, and tyrosine residues. Bonferroni-corrected two-sided *p* <0.05. The *x* axis corresponds to the OR for overlapping pathogenic missense variants. Values greater than 1 indicate pathogenic missense enrichment at codons of detected residue types. Error bars represent 95% CI.

Our next goal was to determine whether the CpD gene set was enriched for clinically important genes, thus testing the hypothesis that CpD labeling can be used to prioritize genes associated with human disease phenotypes. We opted to pursue two complementary analyses to test this hypothesis, comparing CpD gene set to monogenic-disease genes found in OMIM^26^ and to a list of FDA-approved drug targets (*n* = 751).^25^ We assembled a curated set of 5,622 unique monogenic diseases associated with 3,974 genes (Table S3). We find that ∼8% (350 out of 3,974) of OMIM monogenic genes have an FDA-approved drug, whereas ∼6% (75 out of 1,243) of the OMIM-CpD genes are established drug targets. Pointing toward opportunities for future drug-development efforts, a more substantial 31% (1,243 out of 3,974) of the OMIM monogenic gene set are also CpD proteins (Figure 1A; Table S3).

Next, we compared the clinical relevance of the CpD protein set to a control set of proteins targeted by FDA-approved drugs. In OMIM, every gene/protein can be associated with any number of clinical-associated phenotypes that are curated from the literature. We posited that a protein with multiple OMIM phenotypes would indicate increased clinical relevance or importance and that the FDA-approved gene set would serve as a positive control and should be well represented among OMIM genes and their curated phenotypes. Consistent with this hypothesis, the FDA-approved drug-target genes make up 20% of the OMIM genes with four or more disease phenotypes but they make up only 7% of the OMIM gene set with one disease phenotype. In contrast, CpD proteins make up between 23% and 33% of the OMIM gene set, irrespective of the number of associated phenotypes. In fact, 33% of the OMIM gene set with a single disease phenotype overlap with CpD proteins. Interestingly, there is little overlap between the OMIM genes that overlap with FDA-approved drugs and those that overlap with CpD (Figure 1A; Table S4). FDA-approved drug-target genes had significantly more OMIM phenotypes than CpD proteins (mean of 1.69 vs. 1.31, respectively, Wilcox test adjusted *p* [*p*_adj_] = 3.7e−11). Genes encoding CpD proteins represented both ends of the OMIM phenotype count spectrum and were mostly highly represented in proteins associated with a single OMIM phenotype (Figure 1B). These differences in the number of OMIM phenotypes associated with CpD or FDA-approved drug targets indicate that CpD detection, while suggestive, is alone not sufficient to assign functional or clinical importance to the labeled protein.

Finally, given the broader range, relative to the FDA-approved drug targets, of representation among OMIM phenotypes associated with CpD proteins, we next asked whether the CpD dataset is significantly enriched for OMIM genes, again working under the hypothesis that detection via chemoproteomics provides a proxy for clinical importance. We tested each CpD amino acid separately, CpD-Cys (proteins with detected cysteine sites), CpD-Lys (detected lysine sites), CpD-Tyr (detected tyrosine sites), and CpD-CysLysTyr (proteins with all three types of sites). We find that the set of all CpD proteins was significantly enriched with OMIM genes, relative to all other human genes (Figure 1C). When compared to a control set of genes, such as the set of homozygous loss-of-function (LoF)-tolerant genes,^37^ we find that, as expected, this control set is depleted of OMIM disease genes, while the FDA-approved drug-gene set is the most highly enriched for OMIM genes. We cannot, however, rule out the contributions of modestly increased protein length between OMIM genes and non-OMIM genes (Figure S2) to our enrichment of CpD in OMIM genes. Thus, we concluded that CpD information can provide novel and orthogonal evidence for clinical variant predictors that are not currently incorporated into measures of functionality or related to disease states.

### CpD genes are highly constrained for missense variation

Constraint is a widely used measure of gene importance that quantifies the degree of gene intolerance to new mutations in the population. Building on our observation that CpD genes are enriched for monogenic-disease genes, we hypothesized that CpD genes might similarly be enriched for a subset of genes that are highly constrained for missense variation. Interestingly, we find that CpD-CysLysTyr proteins had the highest odds for missense gene intolerance (OR = 6.794, *p* = 1.49e-89) compared to all other human genes (Figure 1D; Table S5), and this enrichment was higher than that for OMIM genes (Figure 1C). Notably, this missense constraint extended to all classes of CpD proteins spanning those captured with cysteine (OR = 3.45, 95% confidence interval [CI] [3.12–3.87]), lysine (OR = 4.02, 95% CI [3.60–4.50]), and tyrosine reactivity (OR = 5.27, 95% CI [4.56–6.09]). Our results support that CpD proteins are even more highly enriched for missense-constrained genes compared with the OMIM genes set (Figure 1D). The set of 114 missense-constrained genes were also enriched for CpDAAs, with 83% (95 out of 114) having at least one CpDAA (Figure S3; Table S6). Further corroborating these findings, this set of 95 CpD genes with missense constraint also showed a high proportion of genes with autosomal-dominant inheritance (Figure S4).

### CpD proteins are enriched for interactions with other proteins

Finally, as all our analyses thus far focused on genetic-based metrics, we also opted to broaden our investigation to consider protein-level indicators of functional importance. As prior work has indicated the number of interactions that a protein is involved in provides evidence of important functional hotspots,^38^^,^^39^ we also evaluated the relative participation of CpD proteins in biological networks compared to all other proteins participation, using annotations from BioGrid, INtAct, DIP, and HPRD.^40^^,^^41^^,^^42^ We find that CpD proteins had significantly more protein-protein interactions compared with all other proteins (Figure S5; Table S7), which provides further evidence of the capacity of chemoproteomics to capture disease-associated and functionally important proteins.

### Detected CpD proteins are enriched for pathogenic missense mutations relative to undetected CpDAA

After establishing that CpD datasets are enriched for functionally important genes, we next sought to understand whether this association would hold true at the residue level. To test our hypothesis that CpDAAs are enriched in functional hotspots, we opted to focus on monogenic-disease-associated genes, given their established clinical importance in disease and a validated set of pathogenic missense variants. To generate a test dataset, we filtered the OMIM monogenic gene set for genes harboring at least one CpDAA residue, as well as at least one pathogenic and one common/benign missense variant. In total, 926 genes fulfilled these criteria, which we will refer to as OMIM&CpD. Within the OMIM&CpD set, 14.12% (1,907 out of 13,503) of cysteine, 4.24% (2,092 out of 49,364) of lysine, and 2.66% (548 out of 20,606) of tyrosine residues have been detected via chemoproteomics (Figure 1E).

Focusing on this OMIM&CpD gene set, we next asked whether CpDAA sites were enriched for pathogenic missense variants. We reasoned that, if CpDAA residues represented a surrogate for functionality, missense mutations at or around these sites could cause monogenic disease. To assess this, we compared the detected cysteine, lysine, and tyrosine (CpDAA-CysLysTyr) positions to the non-chemoproteomic-detected sites (non-CpDAA-CysLysTyr) within the OMIM&CpD set. In the OMIM&CpD set that directly overlaps with missense alleles, ∼1.9% of CpDAA-CysLysTyr positions overlap pathogenic missense alleles compared to only 0.9% of positions that remain undetected by chemoproteomics (Figure S6). CpDAA-CysLysTyr positions were significantly enriched over non-CpDAA-CysLysTyr positions for pathogenic missense alleles (OR = 1.54; *p* = 1.75e−04) (Figure S7). In contrast, CpDAA-CysLysTyr positions were significantly depleted over non-CpDAA-CysLysTyr positions for background missense allele overlaps (OR = 0.89, *p* = 1.56e−04). These findings support that CysLysTyr detection could prove potentially useful for interpreting variant deleteriousness. However, we cannot entirely rule out potential confounding factors that also contribute to CysLysTyr detection, such as amino acids that are found in peptides not well suited to chemoproteomic detection (e.g., long or short tryptic peptides).

As the OMIM&CpD set consists of labeling data for three amino acids, cysteine, lysine, and tyrosine, we next further stratified the variants based on amino acid type. This analysis revealed that CpD-Lys positions relative to non-CpD-lysine positions showed the greatest odds for pathogenic missense overlap (OR = 2.56, *p* = 8.08e−05), followed by CpD-Tyr positions (OR = 2.56, *p* = 8.08e−05) relative to non-CpD-tyrosine positions (Figure 1F; Table S10). CpD-Cys positions showed positive but no significant associations with pathogenic allele overlap (OR = 1.341, *p* = 0.0754) relative to non-CpD-cysteine positions (Figure 1F). These data suggest chemoproteomic residue-specific differences in terms of functional importance, with lysine and tyrosine possibly being more strongly associated with functional significance. Alternatively, these trends could also be indicative of broader genome-wide differences between the three CpDAA residue types.

### Genes associated with monogenic disease are depleted of cysteine amino acids relative to non-monogenic-disease genes

As cysteine and tyrosine are relatively rare amino acids compared to lysine, we next considered whether amino acid content could shed light on the aforementioned chemoproteomic residue-specific differences. We first determined whether OMIM genes (*n* = 3,744) had differences in the mean abundance of amino acid compositions relative to all other genes (*n* = 13,543) that had not been implicated in monogenic disorders (Figure S8A) after correcting for protein-length differences (Table S8). We find that OMIM genes are significantly depleted of cysteine residues (Figure S8A). This trend was consistent across both cysteine codons, ruling out codon specific effects (Figure S8B; Table S9). Many of the cysteines that are clinically pathogenic are part of disulfide bonds,^43^ and chemoproteomics experiments often fail to detect cysteines involved in structural disulfide bonds due to experimental setup. Therefore, this lack of disulfide detection may, in part, rationalize the negligible to minimal cysteine enrichment at pathogenic mutation sites (Figure 1F) despite the known abundance of pathogenic missense mutations.^44^

We also considered the amino acid and codon content more broadly to determine whether these trends were unique to cysteine. Consistent with prior work that revealed depletion of arginine in the context of cancer and health genomic variation,^22^ we also find that two specific arginine codons (AGG and AGA) are similarly highly depleted, whereas other codons are less substantially different between OMIM and other tested gene sets. Intriguingly, glycine’s GGC codon was the most enriched codon in OMIM genes (Figure S8B). We expect that these differences stem from a variety of factors, including mutability and the evolutionary age of each amino acid, as reported previously.^45^^,^^46^

### Spectrum of pathogenic missense substitution across OMIM genes

To further understand the spectrum of pathogenic missense substitution, we quantified the substitution frequency between all possible amino acids in OMIM genes, including both CpDAAs and undetected residues, and assessed the differential frequency of pathogenic missense variants compared with background variation (Figure S9). We find that the most frequent pathogenic substitutions are from glycine to arginine (G > R) and leucine to proline (L > P), consistent with the findings of previous studies.^44^^,^^47^ Consistent with prior reports,^44^ loss of cysteine is also prevalent at pathogenic sites, with frequent mutations to tyrosine and arginine. These findings are particularly striking given the prevalence of gain-of-cysteine mutations in both healthy and cancer genomes, as reported previously.^36^^,^^48^

To determine whether specific codons are disproportionately mutated at pathogenic sites, we next calculated the enrichment of pathogenic variants compared with background variants for every possible codon for loss of and gain of cysteine (Figure S10A), lysine (Figure S10B), and tyrosine (Figure S10C). Both cysteine codons are similarly enriched for pathogenic loss-of-cysteine mutations across all possible substitutions, indicating an absence of codon bias (Figure S10A). The largest enrichment is observed for cysteine to phenylalanine’s TGC>TTC codon exchange (OR = 4.875, *p* = 4.57e−60). In contrast to cysteine loss, mutations involving cysteine gains (Figure S10A; right panel) were not all enriched in the pathogenic category, with two out of the four possible serine to cysteine (S>C) exchanges shown as depleted in pathogenic alleles (TCC>TGC OR = 0.52, *p* = 1.39e−06; TCT>TGT OR = 0.33, *p* = 1.53e−14). Looking beyond cysteine, we were intrigued to observe that, for lysine and tyrosine codons, exchanges that result in loss of each amino acid did not show similar enrichment for pathogenic mutations (Figures S10B and S10C). These findings highlight that tyrosine missense variants that transition to or from cysteine are the most enriched for pathogenicity (Figure S10C). Furthermore, for cysteine, the marked enrichment for pathogenic, loss-of-cysteine missense variants, together with the global depletion of cysteine in OMIM genes, highlight the unique genetic and chemical properties of cysteine, with relevance for both drug development and genetic diagnosis.

### 1D relationships between CysLysTyr positions and pathogenic missense variants in OMIM proteins

**Figure 2:**
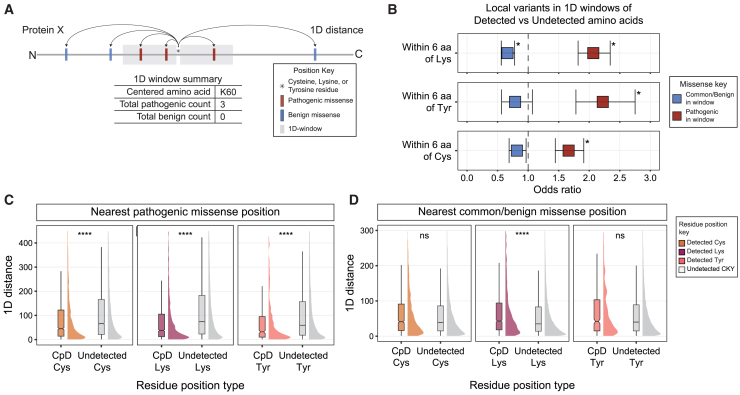
Local enrichment of pathogenic missense variants in 1D space around CpDAA (A) Schematic of 1D distance calculations between CysLysTyr residue positions to positions of missense variants. For each missense variant, the absolute number of amino acids located away from the CysLysTyr position was calculated. A sequence window centered on a reference CpD-CysLysTyr position is shown as a gray box in the toy example. An example summary table shows the total number of unique missense variants within a sequence range of lysine position 60 (K60) in protein X. (B) Odds of pathogenic and common/benign missense variants in 1D windows of detected versus undetected CysLysTyr residues for 926 OMIM&CpD proteins. Error bars of points reflect 95% CI for missense within ±6 amino acid windows of reference CysLysTyr positions. Odds greater than 1 indicate enrichment of specific missense category in detected residue windows. Odds less than 1 indicate enrichment of specific missense category in undetected residue windows and depletion for detected residue windows. Overlaps between missense variant positions and window reference CysLysTyr positions were excluded from the analysis. Bonferroni-corrected two-sided *p* <0.05 calculated by Fisher’s exact test, ^∗^*p* < 0.0083. (C) 1D distances between pathogenic missense positions and detected vs. undetected cysteine (left), lysine (middle), and tyrosine (right) positions. Wilcoxon test for mean comparison with false discovery rate (FDR) adjustment of *p* values. ^∗∗∗∗^*p* < 2e−16. (D) 1D distances between common/benign missense positions and detected vs. undetected cysteine (left), lysine (middle), and tyrosine (right) positions. Wilcoxon test for mean comparison with FDR adjustment of *p* values. ^∗∗∗∗^*p* = 2.8e−11; ns, not significant; cysteine *p* = 0.084; tyrosine *p* = 0.150.

As our dataset of pathogenic missense variants at CpD codons was comparatively small and, as a result, somewhat underpowered (Figure S6), we next opted to step beyond specific mutation sites and began to consider the protein regions surrounding CpD sites. Our goal was to determine whether CpD detection proximal to genetic variants could provide insight into the potential functionality of missense variants outside of the directly impacted and pathogenic residue. To test this hypothesis, for all OMIM&CpD proteins, we analyzed the CysLysTyr residue landscape in 1D linear sequence space (Figure 2A). We measured the relative amino acid distance between CpDs and proximal variants, defined here as “windows.” For example, a distance of zero indicates a direct overlap between CysLysTyr residue and missense alleles, while a distance of three equates to three amino acids separating CysLysTyr from the missense variant.

As a matched control, we generated distance calculations for all non-detected CysLysTyr residues in the same gene set. In this analysis, we did not include any CpD that directly overlapped with a pathogenic missense variant (a distance calculation of 0), which represented a small fraction of each CpD set: 2.4% of CpD-Cys, 1.2% of CpD-Lys, and 2.7% of CpD-Tyr (Figure S6). Since we showed that chemoproteomic-detected residues were significantly enriched among pathogenic variants (Figure S7), we were concerned that the inclusion of directly overlapping sites might inflate the proximal effects of variants.

To further understand this enrichment, we next focused on the immediate neighborhood around the CpDAA, as defined by a six-amino acid window. We observed a significant enrichment of pathogenic missense variants within six amino acids: CpD-Lys (OR = 2.066, *p* = 3.40e−26), CpD-Tyr (OR = 2.221, *p* = 6.49e−12), and CpD-Cys (OR = 1.663, *p* = 3.46e−12) (Figures 2B and 2C). Common benign variants were modestly, albeit significantly, depleted only for CpD-Lys residues (Figures 2B and 2C). Further corroborating the enrichment of pathogenic variants near all CpDAA types, we also find that these 1D windows around CpDAA contained more pathogenic missense variants compared with non-functional variants. 15.6%–20.8% of CpDAA 1D windows include positions of pathogenic missense alleles compared to only 7.9%–9.2% of 1D windows for common/benign missense positions (Figure S11). Surprisingly, missense VUSs were present in nearly half of the CpDAA windows (Figure S11), which suggests that some of these VUSs are damaging mutations that are yet to be functionally characterized.

Consistent with our hypothesis, across all CpDAAs, we found that the distance to pathogenic missense variants was shorter than the common/benign variant set (Figure S12). Further corroborating our hypothesis, by anchoring on either pathogenic or common/benign missense variants, we found that CpD-CysLysTyr residues were significantly closer than undetected CysLysTyr positions to pathogenic missense positions (Wilcoxon test, *p* < 2e−16; Figure S13) in OMIM&CpD proteins. In contrast, common/benign missense positions were significantly closer to undetected CysLysTyr positions than to CpDAA-CysLysTyr positions (Wilcoxon test, *p* = 3.6e−10; Figure S13).

As pathogenic variants were generally enriched in proximity to CpDAAs (Figure 2B), we next opted to stratify our analysis further to consider each CpDAA type and the distance calculated to the nearest groups of missense variants. Consistent with the CpDAA-CysLysTyr analysis, CpD-Lys, CpD-Tyr, and CpD-Cys residues were all found to be significantly closer to pathogenic missense positions than their non-CpDAA counterparts (Figure 2C). No significant differences were detected in the distances to common/benign missense variants between CpD-Tyr and undetected tyrosines or CpD-Cys and undetected cysteine residues (Figure 2D). CpD-Lys detected residues that showed significantly further from common/benign missense variants than undetected lysine residues (Figure 2D, middle panel, *p* = 2.8e−11). Thus, we conclude that pathogenic missense variants are significantly closer to CpD-Cys, CpD-Tyr, and CpD-Lys compared with matched undetected amino acids. Consistent with this, there is either no significant difference or a significantly further distance between common/benign missense variants and any of the CpDAA in our study.

One possible confounder in our distance-based analysis is that the allele-to-CpDAA distance is influenced by protein length and the number of pathogenic and common/benign missense variants (Figure S14). We performed a comprehensive analysis using different protein-length bins for OMIM&CpD proteins, all sequences, short sequences, and long sequences (Figure S15), and found that pathogenic variants were closer to the detected residues for all OMIM&CpD proteins. Taken together, this analysis revealed that CpDAA sites and pathogenic missense variants were significantly closer to each other than common missense variants and undetected amino acid sites, respectively.

### Pathogenic missense variants are enriched in close 3D proximity to CpDAA

Linear protein sequence does not fully capture the microenvironment surrounding the residue of interest. Therefore, we extended our proximity analysis to consider the 3D protein environment of detected CysLysTyr residues. Using our established pipeline,^15^ we mapped the CpDAA positions to protein structures available in the PDB. Crystal structures were available for 419 of the 926 OMIM&CpD proteins (Table S11). We then quantified the number of CpDAA side chains proximal (within 8 Å) to either pathogenic, common/benign, or VUS variants. Collectively, CpDAAs were enriched with proximal pathogenic variants compared to common/benign or background missense variants, which were depleted within 8 Å of CpDAAs (Figure S16).

**Figure 3:**
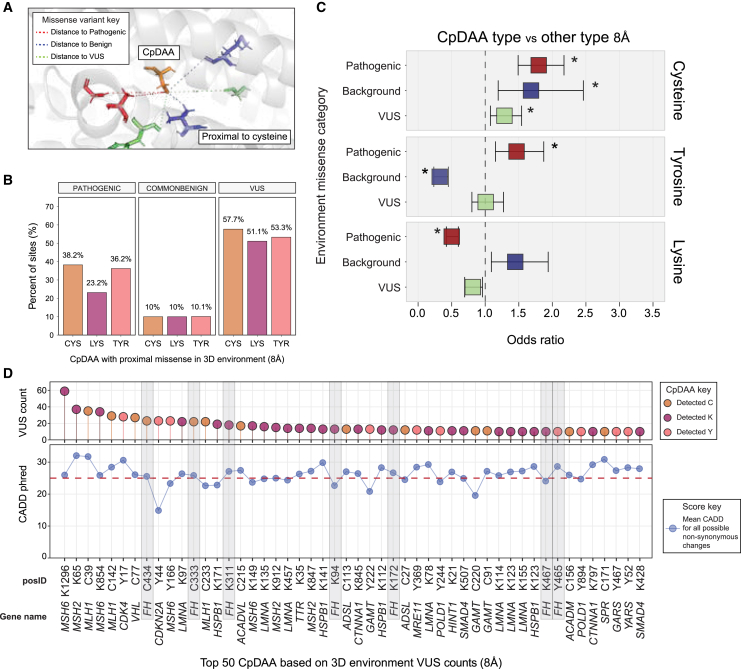
VUS missense alleles are prevalent in local 3D environments of CpDAA residues (A) Cartoon of an example CpD-Cys 3D protein environment. The measured distances between missense variant impacted positions, and CpDAA were based on the terminal atom of CpDAA residues to the nearest atom of neighboring residues for an environment size 8 Å. The distances are shown in the cartoon by dashed lines, with colors corresponding to missense categories: pathogenic (red), background (blue), and VUS (green). Residue positions in the protein structure that overlapped missense alleles are also colored by missense categories. (B) Proportion of CpDAA environments with at least one local pathogenic (left), common/benign (center), or VUS (right) missense variant position for 8 Å sized environments. A total of 419 OMIM&CpD proteins are represented by the data used to create the figure. (C) Significant associations (Bonferroni-corrected two-sided *p* <0.05) calculated by Fisher’s exact test for missense categories that are within 8 Å of specific CpDAA types versus all other CpDAA types included in this study for 419 OMIM&CpD proteins. The *x* axis corresponds to the OR for a particular CpDAA type with a proximal missense variant within 8 Å of their terminal atoms. Values greater than 1 indicate enrichment of proximal pathogenic (red), background (blue), or VUS (green) missense variants for detected cysteine (top, *n* = 570), tyrosine (middle, *n* = 287), or lysine (bottom, *n* = 1,209). Error bars represent 95% CI. (D) Top 50 CpDAA positions based on local missense VUS counts within the 8 Å environment. The top lollipop plot is arranged by decreasing VUS counts within CpDAA environments. The lower plot shows the mean CADD phred scores for all possible non-synonymous substitutions for each CpDAA codon. The deleterious threshold of 25 is marked by a horizontal dashed red line in the plot. CpDAA residues with scores above this threshold were considered to be important for stratification. The CpDAA position IDs for both panels are shown on the *x* axis where C = cysteine, K = lysine, and Y = tyrosine and the amino acid position is indicated after the amino acid.

To obtain a more nuanced understanding of the unique features of the CpD-Cys, CpD-Lys, and CpD-Tyr, we next stratified the CpDAA 3D analysis by amino acid type. We find that CpD-Cys and CpD-Tyr harbor more proximal pathogenic variants than CpD-Lys, with 38.2% (266 out of 696) of CpD-Cys, 36.2% (127 out of 351) of CpD-Tyr, and 23.2% (359 out of 1551) of CpD-Lys being located within 8 Å of a pathogenic missense variant (Figure 3B). In contrast, all three CpDAA neighborhoods were comparatively depleted of common/benign missense variants, with only ∼10% within 8 Å of this variant (Figure 3B). Significance analysis further corroborated these observations, with both CpD-Cys and CpD-Tyr environments enriched for proximal pathogenic missense variants relative to all other CpDAA types (Figure 3C). In contrast, CpD-Lys environments were significantly depleted in proximal pathogenic variants (Figure 3C). The latter observation that CpD-Lys environments were depleted of pathogenic alleles is particularly intriguing, given that the codons were observed to overlap with pathogenic alleles in 1D space. CpD-Cys environments were uniquely enriched for local background missense and VUS missense alleles (Figure 3C), with 58% harboring at least one local missense VUS (Figure 3B). Taken together, these findings again reveal that CpD detection captures functionally relevant sites on proteins, as indicated by the proximity to pathogenic variants, and that cysteines are distinguished by unique attributes when compared to other CpDAAs.

### VUSs are enriched in close 3D proximity to CpDAAs

Given the prevalence of VUSs in our dataset, particularly in proximity to cysteines, we next opted to test whether VEPs^13^^,^^49^ could be used synergistically with chemoproteomic data to gain further insights into the likelihood of a VUS’s potential effect on protein function. We assessed the CADD phred score for each CpDAA. Corroborating the utility of CADD here and consistent with our aforementioned analysis of known pathogenic variants (Figure 3C), we find that CpD-Cys and CpD-Tyr were significantly enriched for pathogenic variants with CADD greater than 25, whereas CpD-Lys did not show such an enrichment (Figure S17).

Therefore, we extended this CADD-based analysis to VUSs. We find that the 50 CpDAA sites with the highest number of proximal VUSs were also more likely to be classified with a mean CADD score greater than 25, indicating an increased likelihood of pathogenicity.^49^^,^^50^ CpDAA environments that are burdened by missense VUSs included lysine 1,296 of the DNA mismatch repair protein MSH6 which had 59 VUSs within 8A of this CpD-Lys (Figure 3D) and lysine 65 of the DNA mismatch repair protein MSH2. Notably, MSH6 and MSH2 form a heterodimer complex as part of their role in the post-replicative DNA mismatch repair (MMR) system.^51^ CpDAA environments of MSH2 and MSH6 lacked local pathogenic alleles based on our ClinVar dataset, whereas the environment of CpDAA residues in MLH1, VHL, and FH proteins had VUS and pathogenic allele-containing environments (Figure 3D; red-colored position ID labels). Taken together, these examples provide evidence that high VUS burden, CpDAA detection, and high CADD score are three metrics that synergize to identify functional sites.

### Cysteine CpDAAs in FH are enriched for pathogenic missense variants

We next opted to identify test cases that could further illustrate the added value of combining chemoproteomics and clinical pathogenicity for capturing functional hotspots. We curated a list of candidate proteins in which there were multiple mapped CpDAAs and could undergo experimental validation of CpDAA function using mutagenesis studies. Our criteria for ideal candidate proteins included (1) the gene has both missense variants in pathogenic or common/benign categories, (2) available PDB structure for protein structure analysis, (3) less than 1,000-amino acid protein length, (4) ClinVar and gnomAD allele position interactions with CpDAA in a 3D environment, and (5) is detected in CysLysTyr proteomic studies. Of the 28 candidate proteins that remained for consideration (Table S12), we performed additional filtering to identify those with known haploinsufficiency, a high number of VUSs, and established functional assays based on expected mutation effects.

Across all candidate proteins, FH stood out. FH is a metabolic enzyme that catalyzes the reversible hydration/dehydration of fumarate to malate in the tricarboxylic acid cycle.^52^ This protein encodes a 510-amino acid protein that forms a tetramer for enzymatic function.^53^ Pathogenic mutations have been shown to be LoF, by either truncating the monomer^54^ or missense mutations disrupting enzymatic function with^55^ or without^56^ disrupted dimerization. These LoF mutations are linked to two distinct OMIM phenotypes, depending on the dosage of the genetic variant, and ClinVar has over 200 VUSs.

The large number of VUS sites and multiple CpD sites in the protein enabled thorough characterization of functional tetramerization at or near CpD sites. Dominant mutations in *FH* (MIM: 136850) are linked to hereditary leiomyomatosis and renal cell cancer (HLRCC) (MIM: 150800).^57^ Individuals with heterozygous *FH* variants have an increased risk of early-onset benign tumors derived from smooth muscle in the skin and uterus as well as an increased risk of aggressive renal cell carcinoma. Individuals harboring recessive mutations have fumarase deficiency (MIM: 606812), a severe neonatal encephalopathy associated with intellectual disability, seizures, and facial dysmorphism.^58^

**Figure 4:**
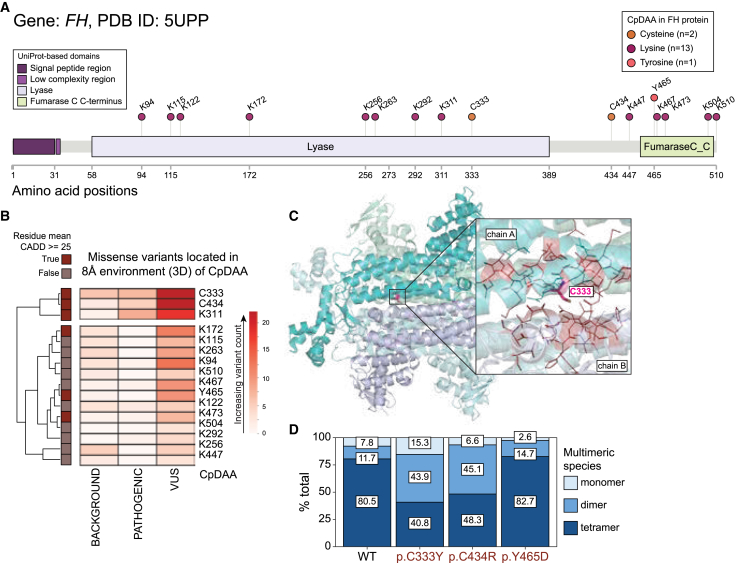
Tetramerization of FH is disrupted by the loss of detected cysteine and lysine residues (A) Schematic of the FH protein with lollipops marking CpDAA positions. Total counts of cysteine (C), lysine (K), and tyrosine (Y) residues are shown in the figure key in parentheses. Protein domains (FH UniprotID:P07954) are shown as colored rectangles (FH = 510 amino acids). (B) Heatmap was supervised based on missense variant counts and average distance from CpDAA in an 8-Å radius around CpDAA in FH. Counts of variants from the background missense variants, pathogenic, and VUS categories (*x* axis) were compared across 16 CpDAAs. Data preprocessing included row-centering and not unit-variant scaling. The two groups resulted from row clustering and are shown by the vertical space separating the top three rows from the other rows. Row annotations for CpDAAs are indicated as the amino acid Cys (C), Lys (K), and Tyr (Y) and the amino acid position. Mean CADD scores greater than or equal to the deleterious score threshold are shown in the outermost left column. (C) Crystal structure of FH (PDB: 5UPP), highlighting Cys333 (C333). The VUS positions are specific to the tested inactivating VUS proximal to Cys333. Chain A is green, and chain B is purple to distinguish interacting domains from two subunits in the tetramer. Images were generated using PyMOL.^59^ (D) Densitometry was used to quantify the percentage of each multimerization species for the *FH* variants in a previous study^56^: p.Cys333Tyr, p.Cys434Arg, and p.Tyr465Asp missense variants that overlap codons of CpDAAs are shown in red text on the *x* axis compared to the wild-type (WT) protein.

Our data resolved 16 CpDAA sites (*n* = 16 CpDAA sites: 2 CpD-Cys, 13 CpD-Lys, and 1 CpD-Tyr) (Figure 4A). *FH* has 410 total non-synonymous SNVs (52 pathogenic, 3 common/benign, 12 rare, 85 rarest, and 258 VUS) that impact 341 unique residue positions of the FH protein. We next counted the number of clinically identified variants (pathogenic, likely pathogenic, and VUSs) that were localized within 8 Å of the CpDAA site. Clustering based on the total number of proximal missense variant counts resulted in a separation into two groups: CpDAA with more than 20 missense variants and CpDAA with fewer. The residue group associated with higher local VUS counts included two CpD-Cys residues (Cys333 and Cys434) and one CpD-Lys residue (Lys311). All three CpDAAs in the top cluster were also associated with deleterious CADD scores for possible residue substitutions by missense variants (Figure 4B).

The cysteine at position 333 in the FH protein was within 8 Å of four pathogenic missense alleles, six gnomAD alleles in the rarest allele frequency category, and 24 missense VUS alleles combined from ClinVar and a validation screen by Wilde et al.^56^ (Figure S18). Position Cys333 of FH is located within an α helix at the tetramerization interface of the FH enzyme and within 8 Å of the CpD amino acid. Of the 44 amino acids within an 8 Å sphere, 77% of the variants (34 out of 44) were classified as pathogenic, likely pathogenic, or VUS, highlighting the enrichment of disruptive variants within 3D space, which are not enriched in linear (1D) space (Figure S18). Finally, to confirm that these CpDAA variants disrupted tetramerization, we performed a gel-based assay using mutated plasmids to assess the efficiency of monomerization, dimerization, and tetramerization. We found that the Cys333Tyr and Cys434Arg variants showed an increased proportion of dimerization compared to wild-type FH (Figure 4D), which further supports the utility of CpDAA detection in pinpointing functional missense alleles.

## Discussion

Here, we brought together human genetic data with proteome-wide measures of amino acid functionality to assess the potential impact of chemoproteomic data in pinpointing functional missense variants. We first started by considering gene-level features, which revealed intriguing associations between CpD detection, increased genetic constraint, enrichment for pathogenic variants, and OMIM phenotypes, corroborating that CpD datasets are enriched for disease-relevant proteins. To assess broader genomic features that could contribute to these relationships, we also more broadly analyzed the patterns of missense substitution for genes in OMIM. These analyses revealed that gain of proline and loss of lysine were strongly associated with pathogenic clinical annotation and that cysteine amino acids were significantly depleted in monogenic-disorder genes relative to all other protein-coding genes. Guided by these findings, we then assessed the proximity of CpDAAs to pathogenic variants, which revealed that, in both 1D sequence and 3D space, protein regions proximal to CpDs are enriched for pathogenic missense variants and VUS. Taken together, our work further corroborates that chemoproteomics detection is a useful metric for discovery of functionally important regions of proteins.

We expect many useful applications of our findings. Chemoproteomic detection may prove particularly impactful for assessing the missense intolerance of smaller genes (containing fewer codons) or highly paralogous genes for which constraint metrics based on observed-over-expected ratios of rare missense variation are less reliable. Lysine-detection annotations may be important for rare-variant interpretation, given that CpD-Lys residues are significantly associated with pathogenic-allele overlap relative to all other lysine positions in monogenic-disorder-associated proteins. Cysteine-detection annotation may serve as an important marker of pathogenic-allele burden regions for genes and proteins based on 1D and 3D spatial analysis of missense alleles. Incorporation of unsupervised machine-learning-based annotations such as the CADD score appears to support stratification of CpDAA and complement analysis of proximal missense-burden analysis of CysLysTyr residue positions.

Our study also highlights many potential areas to further grow the synergisms between genetics and chemoproteomics for drug-development applications. To date, less than 5% of rare diseases have FDA-approved drugs.^60^ Our results revealed that one-third of the rare disorder-associated proteins had cysteine, lysine, and/or tyrosine-reactive sites, which represent intriguing starting points for future drug discovery campaigns. While these sites are highly intriguing given the burgeoning enthusiasm for covalent drug discovery, we do acknowledge that some monogenic genes may prove ill-suited as drug targets due to mimicry of clinical diseases. This limitation is in a manner akin to the recently observed correlation between phenotypes associated with monogenic disorder-associated genes and clinical trial side effects.^61^

We do also acknowledge some limitations of our work, which we hope can inspire future studies. The chemoproteomic datasets harnessed here cover only a fraction of all theoretically detectable CpDAAs. With the substantial recent advances in mass spectrometry technology, we expect that this coverage gap will begin to narrow, which will further power future studies, including across additional dimensions of data (e.g., cell type or state, environmental conditions), which could further delineate functional sites. ClinVar is also not a “gold-standard,” database as it is uncurated, dependent on clinical laboratories to upload data and interpretation, and many of the variants have conflicting interpretations between clinical labs as our reference data have evolved over the years. Thus, the use of other curated datasets, such as ClinGen,^62^ could further enhance future analyses should it be expanded to all disease genes. We note that these data do not assess every possible variant, such as those developed in multiplexed assays of variant effect, but instead serve as a complementary tool to identify functional and druggable regions of clinically relevant proteins. The limited coverage of proteomic sites means that it is not appropriate for screening for pathogenic variants, as the potential for false negatives is high. However, identifying a positive site or region can add to the evidence of pathogenicity. Lastly, this study focused on only a subset of human genes, namely those validated as causal in rare monogenic disorders; therefore, conclusions may not apply to larger sets of reactive and probe-detected sites in human proteins. Thus, we look forward to testing the relationship between CpDAA reactivity and the severity of missense pathogenicity, using additional complementary high-throughput methods (for example, CRISPR-based editing technology as well as deep mutational scanning).

Taken together we hope that our study will help guide future efforts to decipher the impact of missense variants on human phenotypes and launch new efforts in the development of therapies targeting disease-relevant variants.

## Data and code availability

The raw and processed data generated during this study are available at https://github.com/ArboledaLab/CKY and the access number for the merged and cleaned chemoproteomics data files are in Zenodo: 10.5281/zenodo.15243173 (https://doi.org/10.5281/zenodo.15243173).

## Acknowledgments

We thank the members of the Arboleda and Backus lab for their insight and feedback throughout this project. Palafox was supported by the Eugene V. Cota-Robles Fellowship and Chemistry Biology Interface Training Program T32GM008496. B.R.W. was supported by a Postdoctoral Fellowship (133839-PF-19-203-01-CCG) from the 10.13039/100000048American Cancer Society. H.C. was funded by R01 CA215185, R01 AR070245, and an Advanced Discovery Award from the 10.13039/100003064Kidney Cancer Association. K.M.B. was supported by DP2 GM146246-02, Packard Fellowship for Science and Engineering (2020-71388), and an Beckman Young Investigator Award from the 10.13039/100000997Arnold and Mabel Beckman Foundation. V.A.A. was supported by 10.13039/100000002NIH award DP5OD024579, a 10.13039/100015591Rose Hills Foundation Innovator Award, and a 10.13039/100000888Keck Foundation Junior Faculty Award.

## Author contributions

M.F.P., V.A.A., and K.M.B. conceived and designed the study. M.F.P. wrote code, analyzed data, and made figures. L.B. contributed to data analysis. H.C. and B.R.W. contributed to data collection and insights into Figure 4. M.F.P., V.A.A., and K.M.B. interpreted the results and wrote the manuscript.

## Declaration of interests

The authors declare no competing interests.

## Supplemental information

Data S1. Tables S1–S3

Document S2. Article plus supplemental information
