# Supplementary material for: Prioritizing disease-associated missense variants with chemoproteomic-detected amino acids
Source: Am J Hum Genet. 2025 May 23;112(7):1649–63. doi: 10.1016/j.ajhg.2025.04.017 (PMC12256893; doi:10.1016/j.ajhg.2025.04.017)
Supplement: Document S1. Figures S1–S18 and supplemental methods [file mmc1.pdf]

The American Journal of Human Genetics, Volume *112*

Supplemental information

Prioritizing disease-associated missense variants with chemoproteomic-detected amino acids

Maria F. Palafox, Lisa Boatner, Blake R. Wilde, Heather Christofk, Keriann M. Backus, and Valerie A. Arboleda

The American Journal of Human Genetics, Volume *112*

Supplemental information

Prioritizing disease-associated missense variants with chemoproteomic-detected amino acids

Maria F. Palafox, Lisa Boatner, Blake R. Wilde, Heather Christofk, Keriann M. Backus, and Valerie A. Arboleda

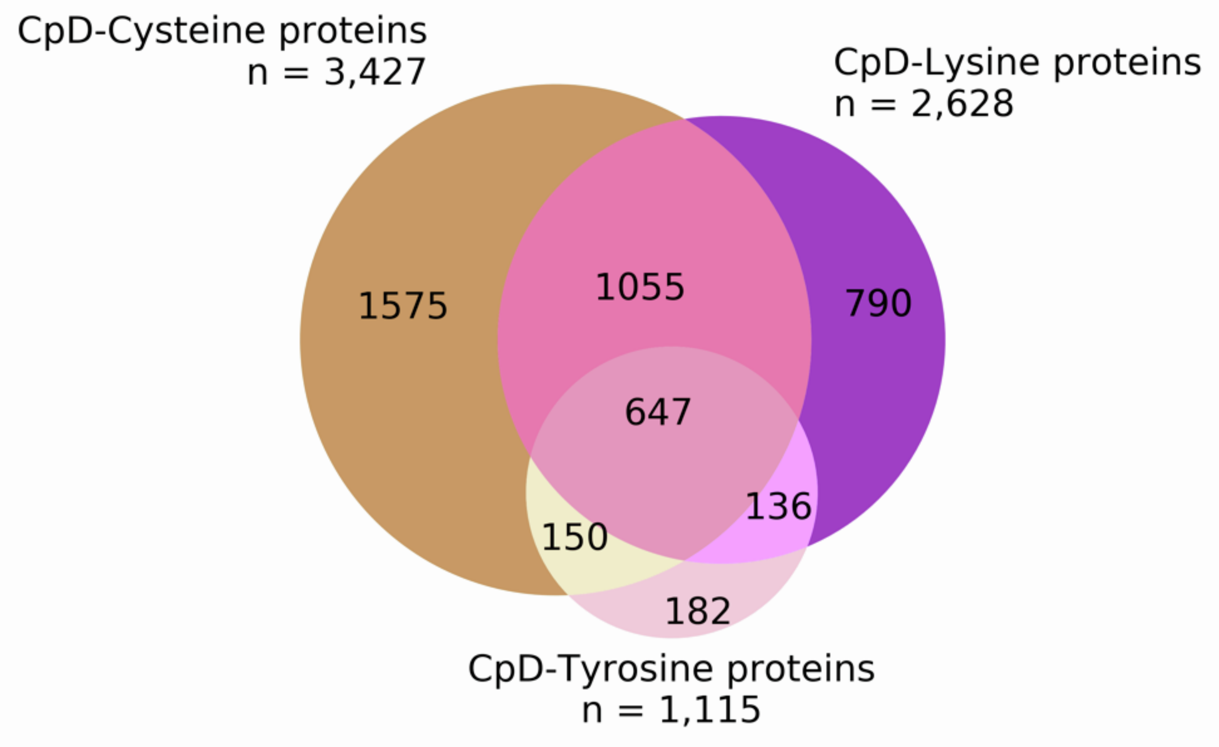

Figure S1. Overlaps between ChemoProteomic-Detected proteins from cysteine, tyrosine and lysine experiments. The venn diagram is based on the merged dataset of 4,535 CpD proteins, with numbers indicating unique UniProt protein identifiers.

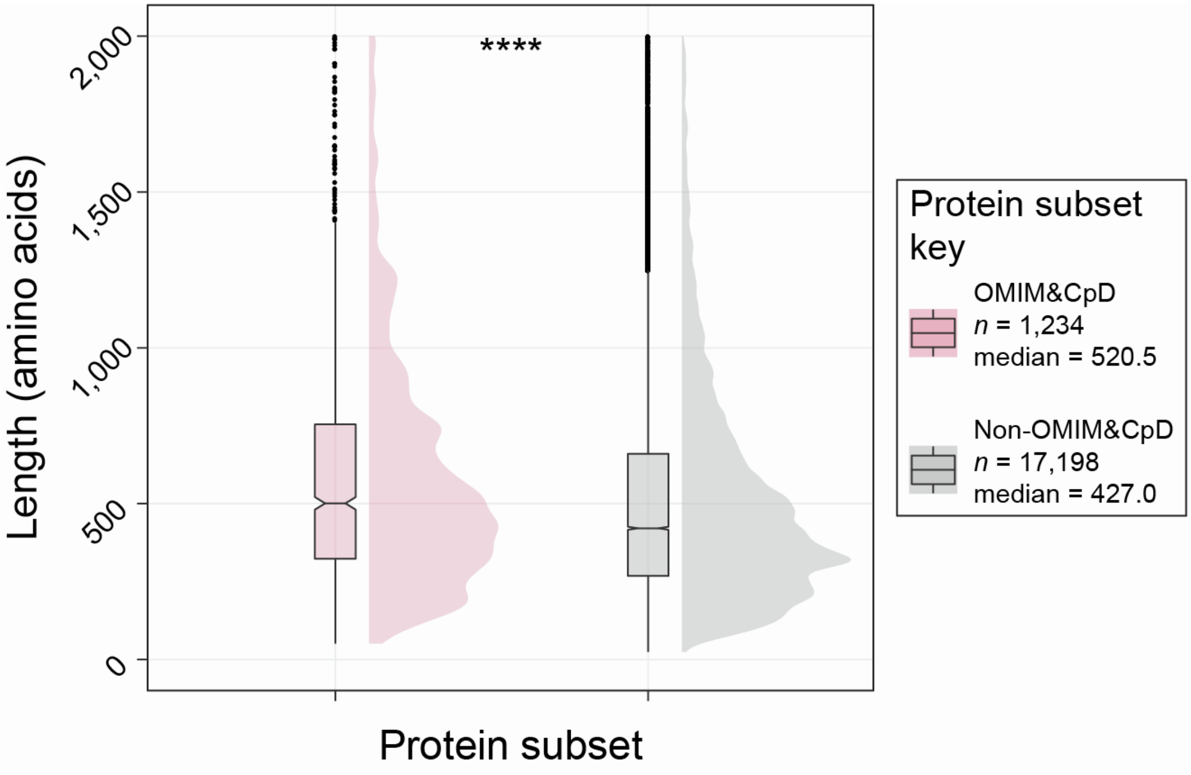

Figure S2. Protein length of OMIM&CpD versus all other proteins. Protein counts and group median values show CpD proteins that are not OMIM genes are on average shorter than CpD proteins that are OMIM genes. Wilcoxon test used for group mean comparison with FDR adjustment of *p* values. ****$p < 2e\text{-}16$. The median length for all human proteins was 434 amino acids. Plot based on 18,432 canonical UniProtKB human proteins.

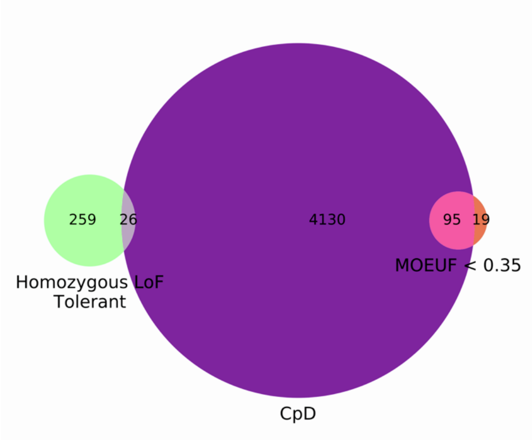

Figure S3. Missense constrained genes show high overlap with CpD genes. Venn diagram shows overlaps of CpD genes with missense constrained genes (n=114; based on MOEUF constraint cut-off < 0.35). We also compared with a control group of genes that are tolerant of variation, termed the homozygous LoF tolerant genes.

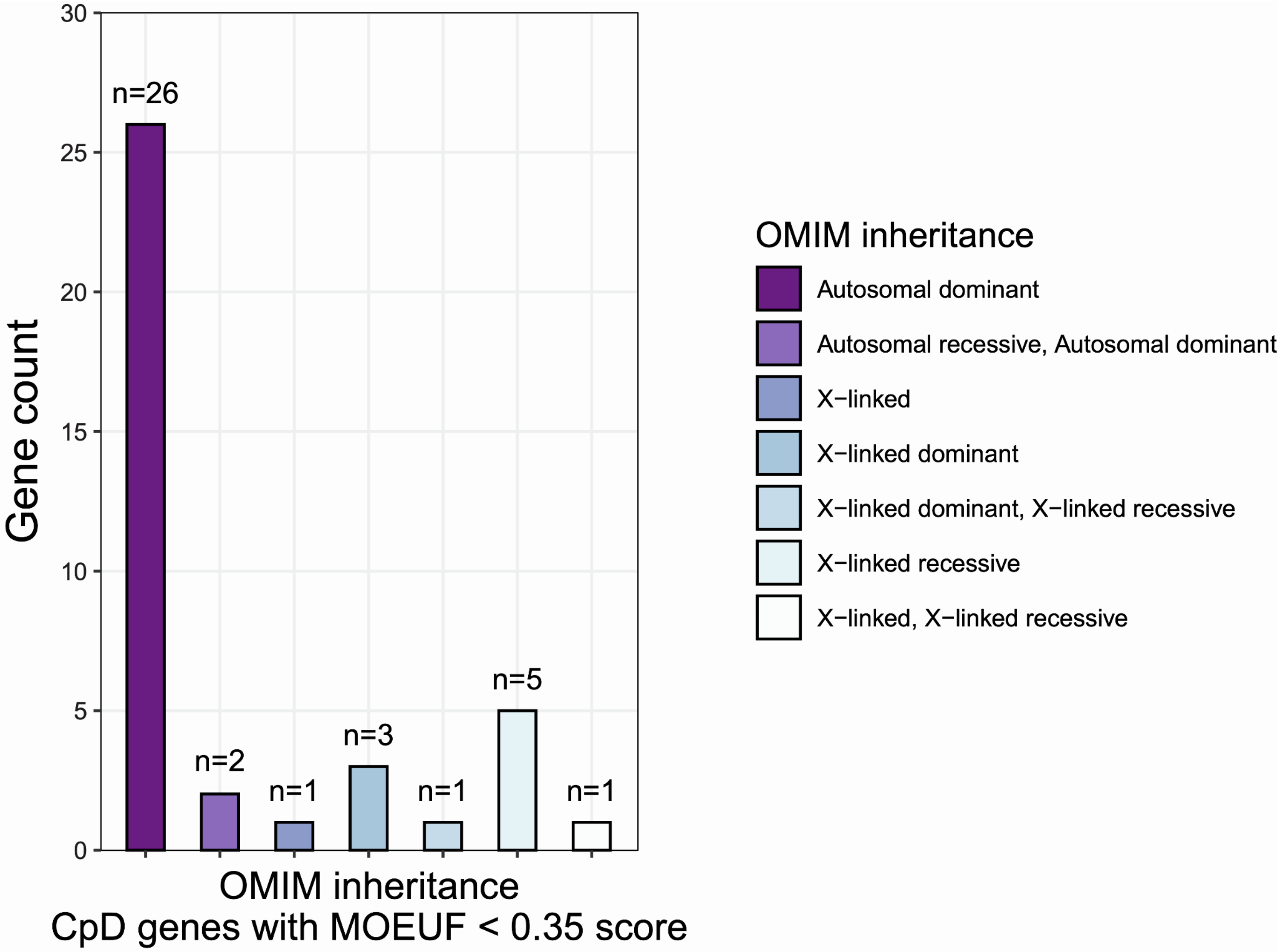

Figure S4. Analysis of OMIM inheritance patterns for single gene disorders in CpD genes with high missense mutation constraint (gnomAD MOEUF < 0.35). From a total of 16,812 genes analyzed, we identified 95 CpD genes with missense constraint, of which *n*=39 had documented phenotype inheritance patterns.

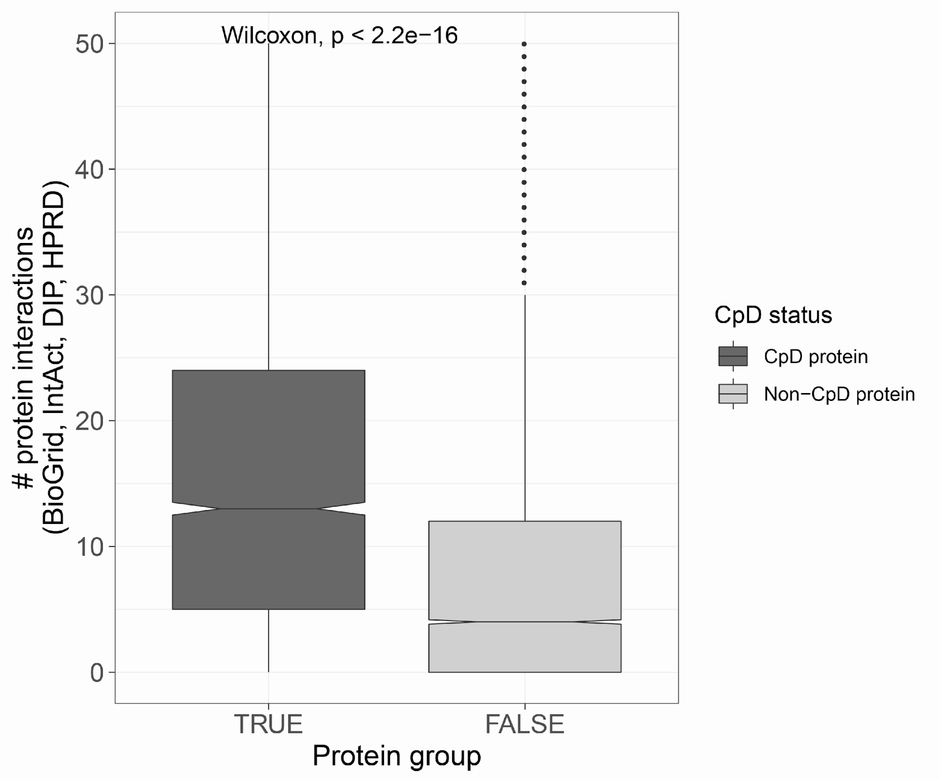

Figure S5. Significant association between CpD as an annotation related to higher interactivity of proteins. The comparison of CpD proteins to all other protein interaction partner counts (BioGrid, IntAct, DIP, HPRD) showed CpD were significantly more connected in biological networks compared to the average non-CpD protein. Based on 4,251 total CpD genes from the universe of 16,812 genes. PPI counts sources from Pei et al. 2021 (**Table S7**).

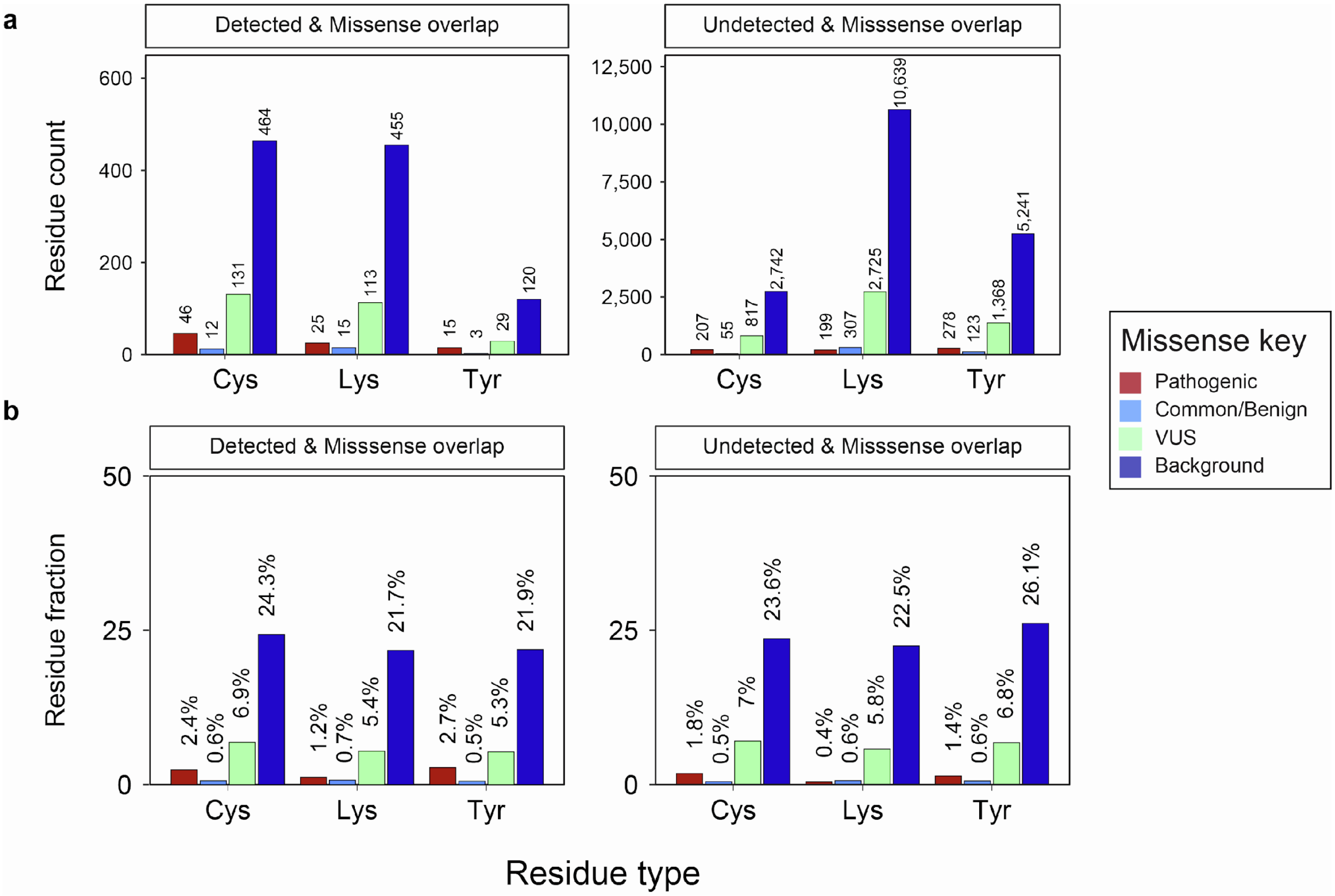

Figure S6. Detected versus undetected CysLysTyr positions overlapping missense variants in OMIM&CpD proteins. a) The counts of CysLysTyr positions overlapping missense alleles; **b)** and the proportion of CysLysTyr positions overlapping missense alleles are based on 926 OMIM&CpD proteins.

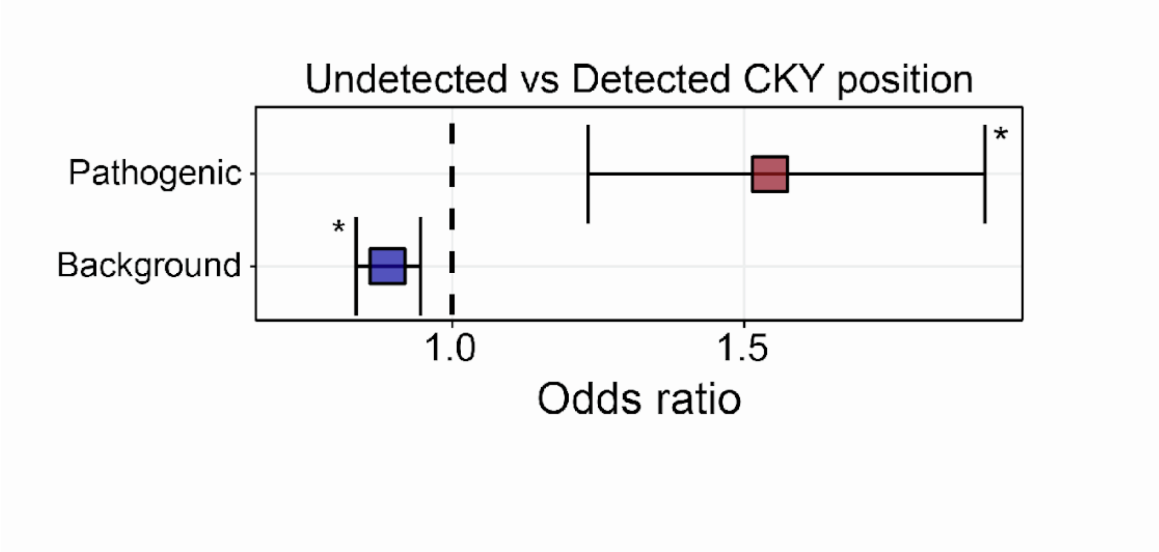

Figure S7. **Detected versus undetected CysLysTyr positions are enriched for pathogenic missense variance compared with undetected CysLysTyr.** Odds of pathogenic and background missense variant overlapping a detected (n = 5,854) versus undetected (n = 304,889) CysLysTyr residue position in OMIM proteins (n = 3,907). Bonferroni-corrected two-sided p value < 0.05 calculated by Fisher's exact test, *p < 0.0042.

A.

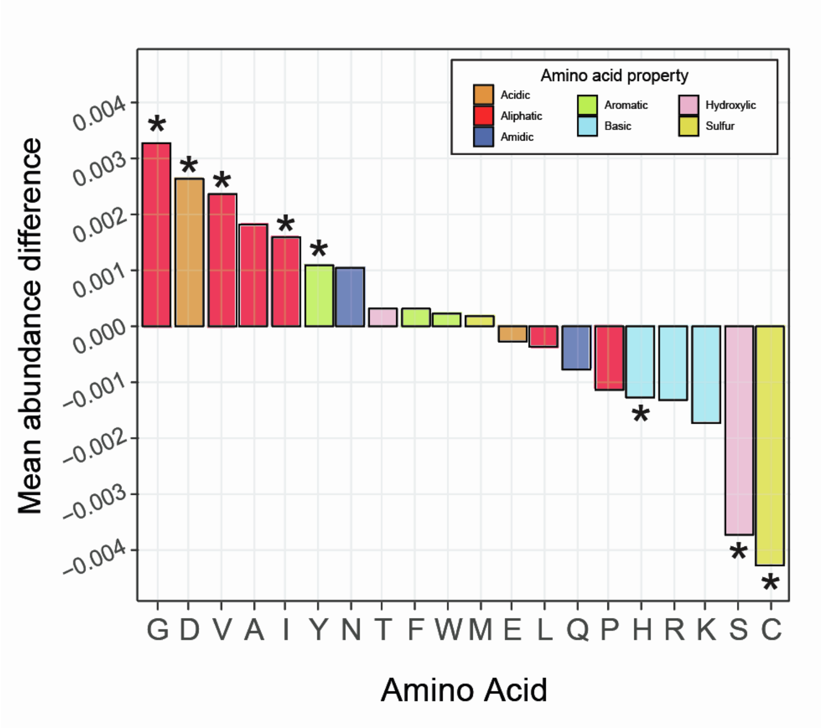

B.

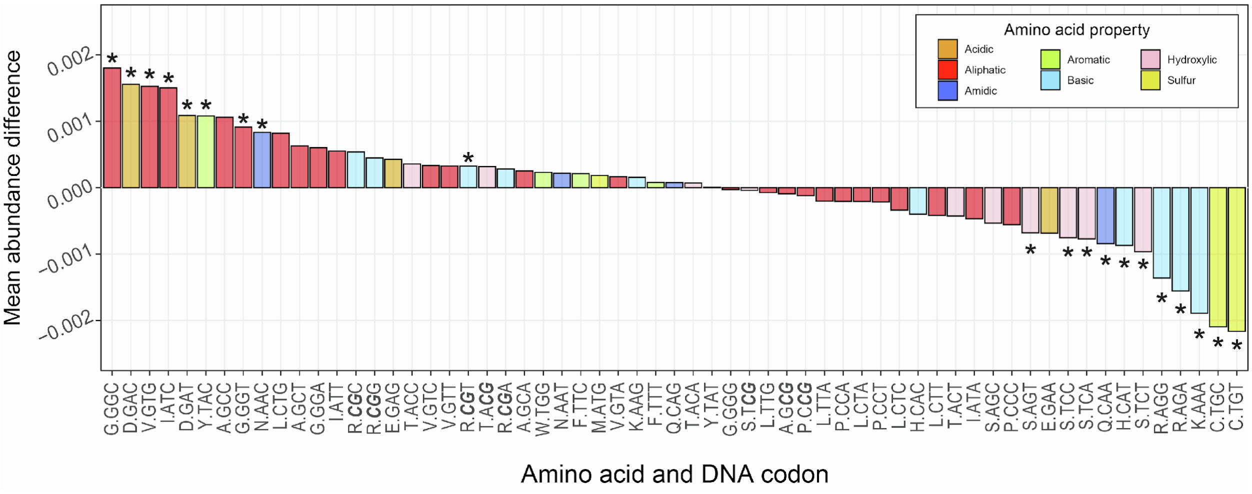

Figure S8. Differences in mean abundance of amino acids and codons in OMIM genes versus all other genes. Total of 17,287 human genes were included in the analysis. **A)** Amino acid (**Table S8**); **B)** Amino acids based on 61 DNA codons (**Table S9**). The codon frequency of each gene was normalized by total codons counted per gene and averaged for all genes in the OMIM and non-OMIM gene sets. The codon labels shown on the x-axis are formatted as the single amino acid letter abbreviation followed by the synonymous DNA codon (i.e. 'C.TGT' for Cysteine's TGT codon). Eight codons on the x-axis contain CpG dinucleotides marked by bold italic font. The y-axis shows mean normalized codon abundance differences. Bar colors are based on physicochemical properties of the encoded amino acid residues. Significant abundance differences were determined using a two-sided, two-sample Welch's t-test and permutation without replacement test. **p*-values < 5.0e-05 in Welch's t test and 1000/1000 permutation instances.

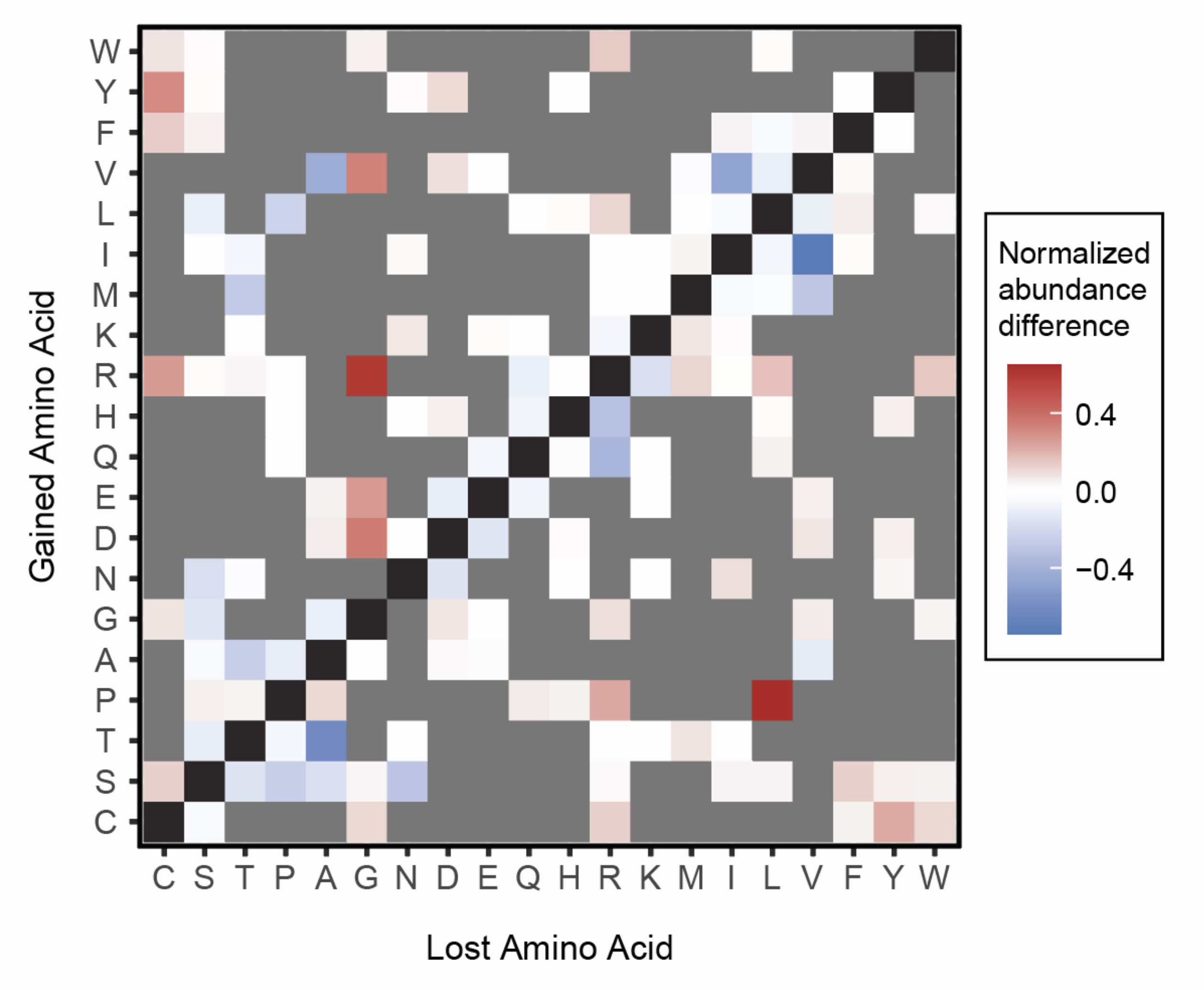

Figure S9. Differential abundance of gain- and loss- of specific missense changes. The magnitude of enrichment for missense involving cysteine in the Pathogenic versus Background missense categories. 95% confidence intervals (line segments) and odds ratios (squares). All possible substitutions by single nucleotide variants were counted, resulting in 3489 Pathogenic and 65505 Background mutations involving a gain or loss of cysteine. Red squares are substitutions enriched in the Pathogenic category and blue squares are substitutions enriched in the Background category. Non-significant odds are shown as transparent squares. Significant Bonferroni-adjusted *p* < 6.38e-05 (two-tailed Fisher's exact test). The analysis was restricted to a subset of 2,873 proteins that have annotations for both categories of missense variant, resulting in a final set of 33,872 Pathogenic and 29,778 Common/Benign variants reelected in the heatmap. The x axis shows the lost (or mutated) amino acid and the y axis shows the gained (or mutant) amino acid. Amino acid single letter labels are ordered based on the side-chain chemistry {Mount DW: Bioinformatics Cold Spring Harbor, NY: Cold Spring Harbor Laboratory Press; 2001}. Substitutions closer to the diagonal line of black squares are considered more conserved, and substitutions farther from the diagonal line are considered less conserved. Pathogenic and Common/Benign missense abundance matrices were normalized so that the sum over all mutation frequencies equals 1.

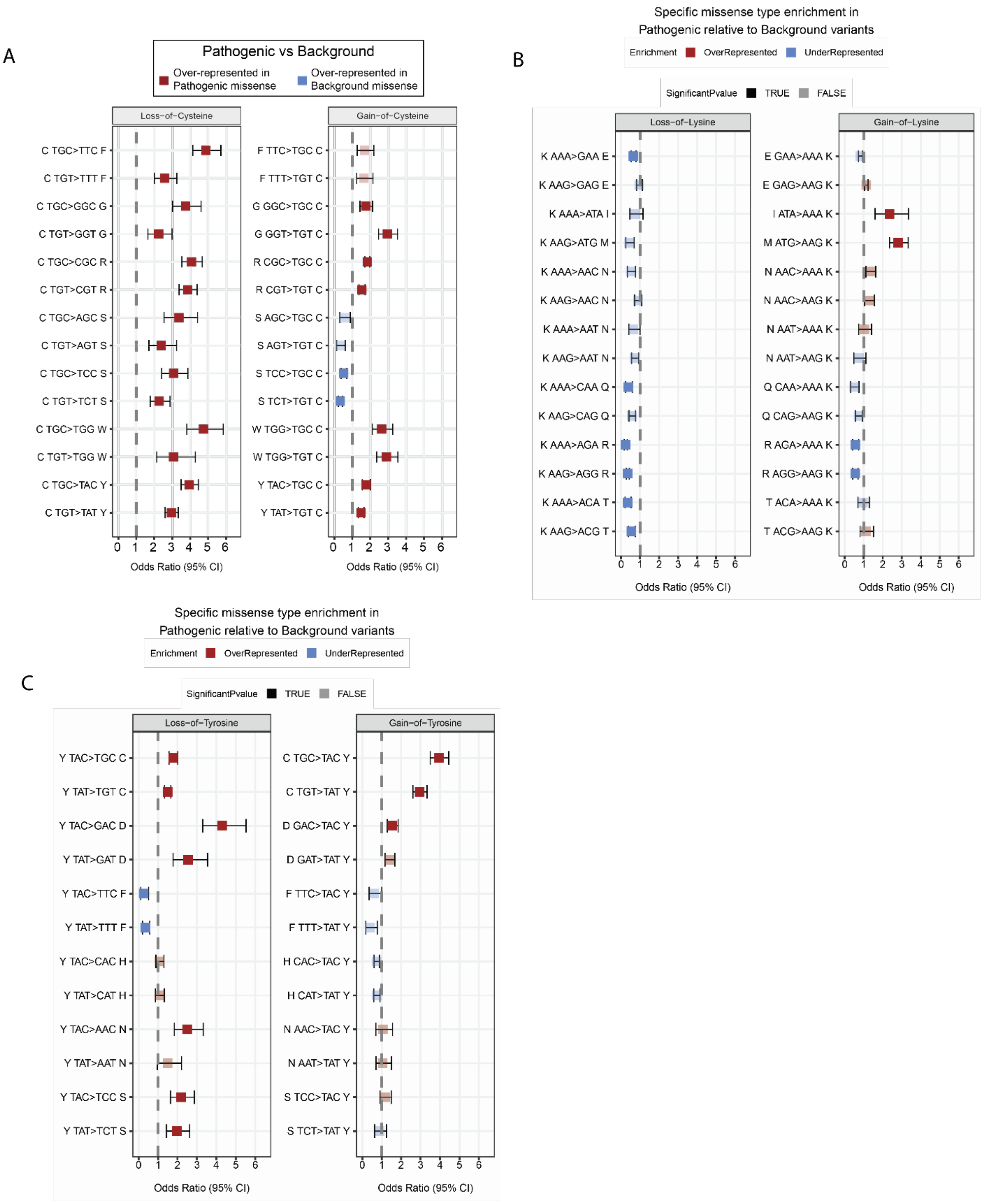

Figure S10. Magnitude of enrichment for pathogenic missense variants versus background missense categories across the three amino acids assessed in our study. All possible substitutions by single nucleotide variants were counted; 95% confidence intervals (line segments) and odds ratios (squares). Red squares are substitutions enriched in the Pathogenic category and blue squares are substitutions enriched in the Background category. Non-significant odds are shown as transparent squares. A) Cysteine; B) Lysine; C) Tyrosine

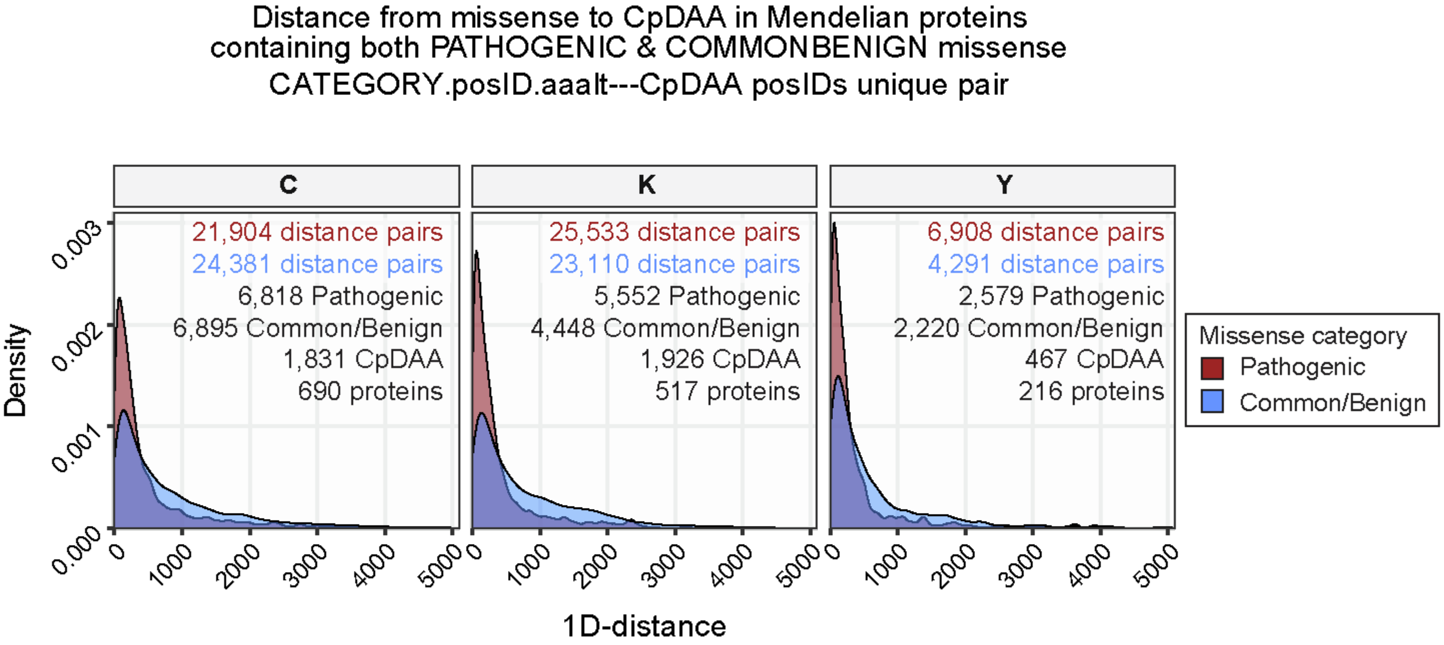

Figure S11. Chemoproteomic-detected amino acids are closer to pathogenic missense than common benign missense. Detected residue distances to pathogenic versus common/benign missense positions in OMIM proteins. Distance distributions are distance pairs based on unique missense positions to unique positions of detected residues. The distributions represent distances between nearest category missense positions to each CpDAA position in the same protein and includes 1D distances of zero, which represent direct overlaps of CpDAA and missense positions.

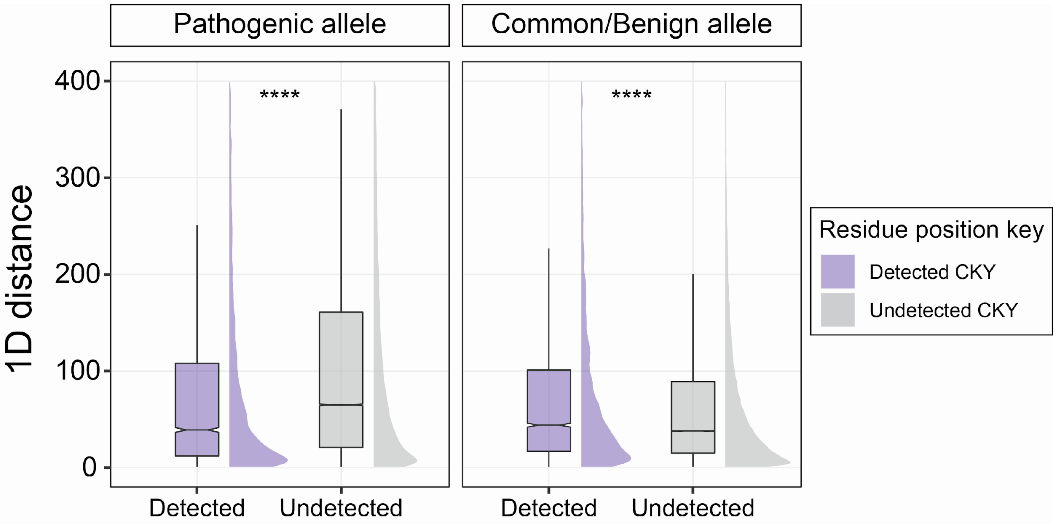

Figure S12. Distance to pathogenic and common/benign missense for detected versus undetected positions. Nearest distances for a given category to a unique CysLysTyr reference position counted for 926 OMIM&CpD proteins. Distances of zero were excluded from the analysis and proteins were controlled to contain at least one pathogenic and one common/benign missense position. Wilcoxon test for mean comparison with FDR adjustment of p values. ****p < 2e-16.

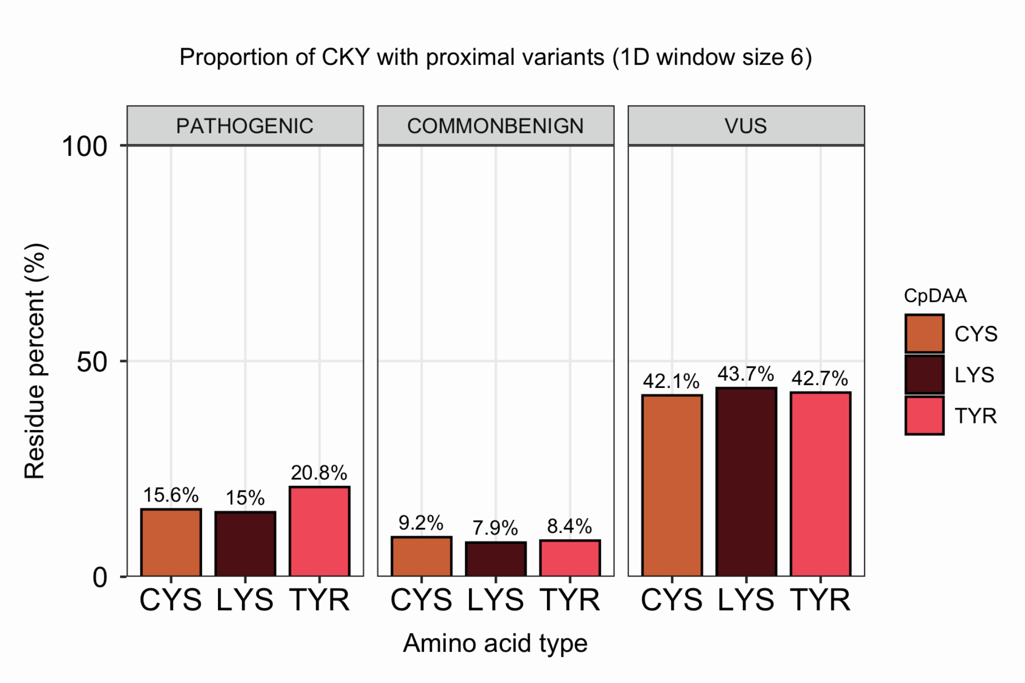

Figure S13. Proportion of detected residue 1D windows with pathogenic, common/benign, and VUS missense alleles. The 1D windows are based on ±6 amino acids from their position in 1D sequence space. Analysis includes 926 OMIM&CpD proteins that have been filtered to contain at least one pathogenic and one common/benign missense variant.

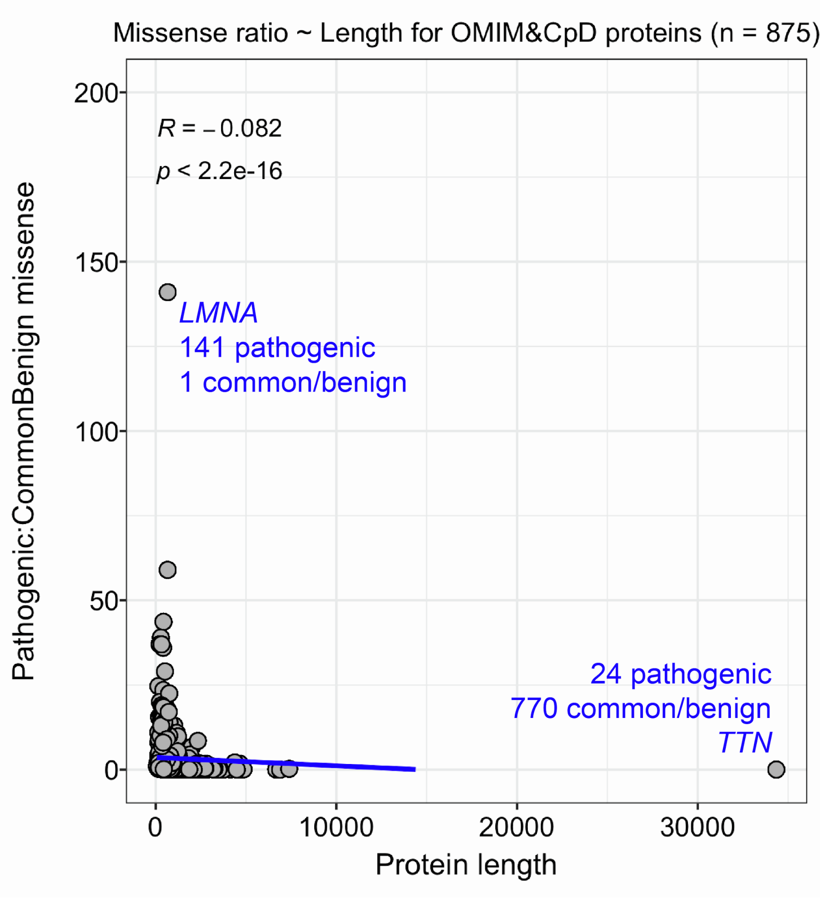

Figure S14. **Ratio of pathogenic:common/benign missense and protein length.** Outlier gene examples are annotated on plot along with the counts of unique pathogenic and common/benign missense per gene.

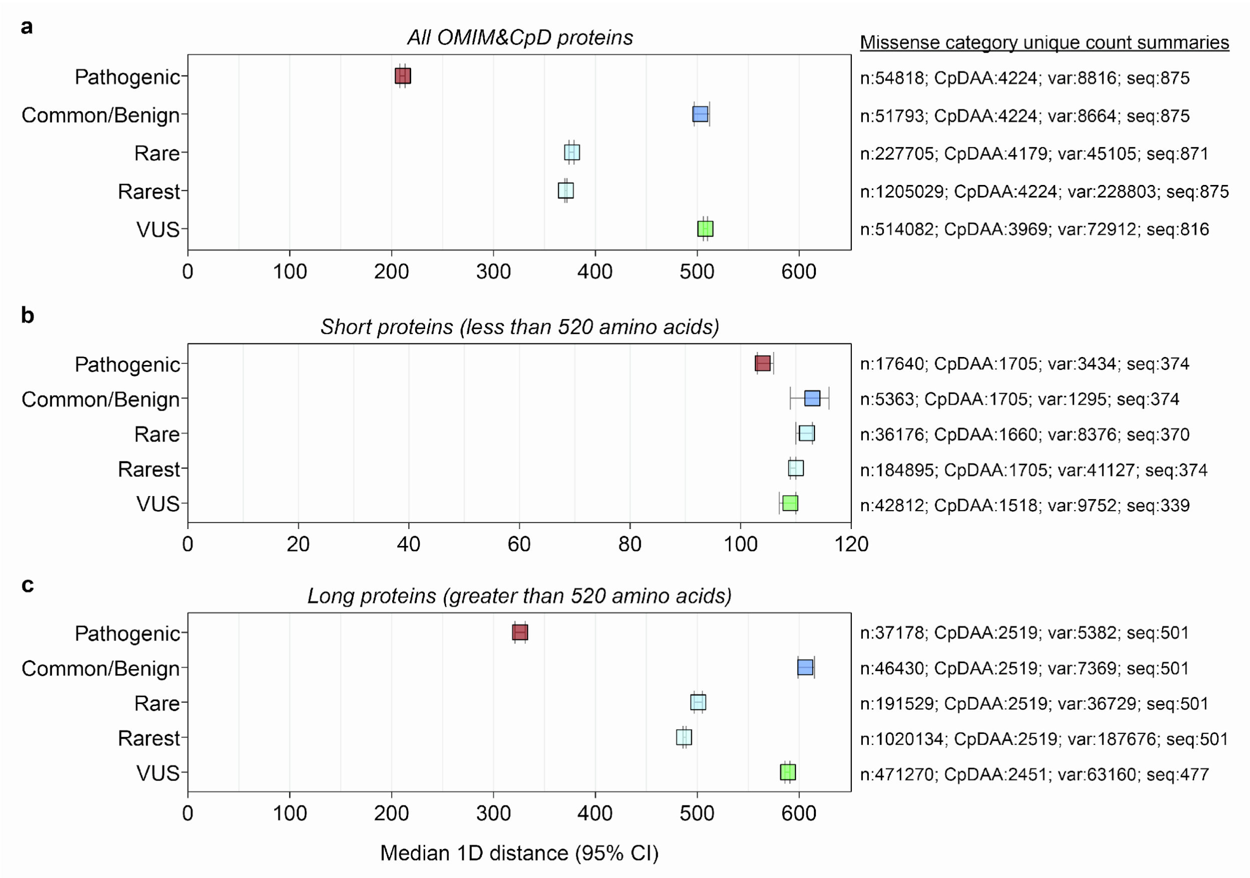

Figure S15. Missense categories to CpDAA-CysLysTyr residues 1D distances. Medians with bootstrapped 95% CI shown for OMIM&CpD proteins. The three subplots show (**a**) all OMIM&CpD proteins, (**b**) short OMIM&CpD proteins, and (**c**) long OMIM&CpD proteins with 1D distance unique count summaries of total distance pairs (n), detected CysLysTyr positions (CpDAA), missense alleles (var), and proteins (seq) for the missense categories (y-axis) shown on the right of each subplot.

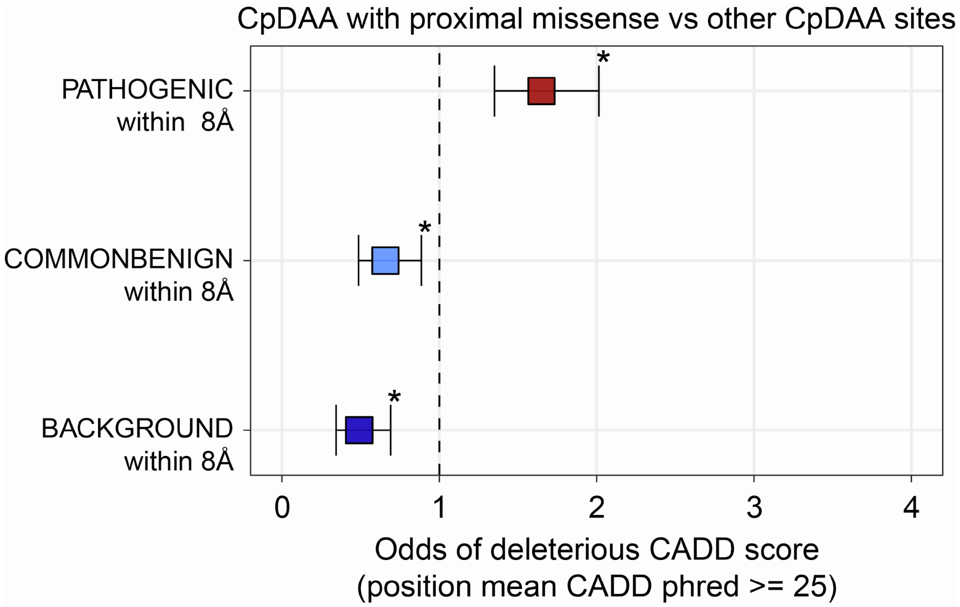

Figure S16. Odds of deleterious CADD score based on missense 3D distance from CpDAA residues. Significant associations calculated by Fisher's exact test for local missense in 3D environment and CADD deleterious scores of all possible substitutions of CpDAA codons. Bonferroni-corrected two-sided p-value<0.05; x-axis corresponds to the odds ratio for 8Å environment; error bars represent 95% CI.

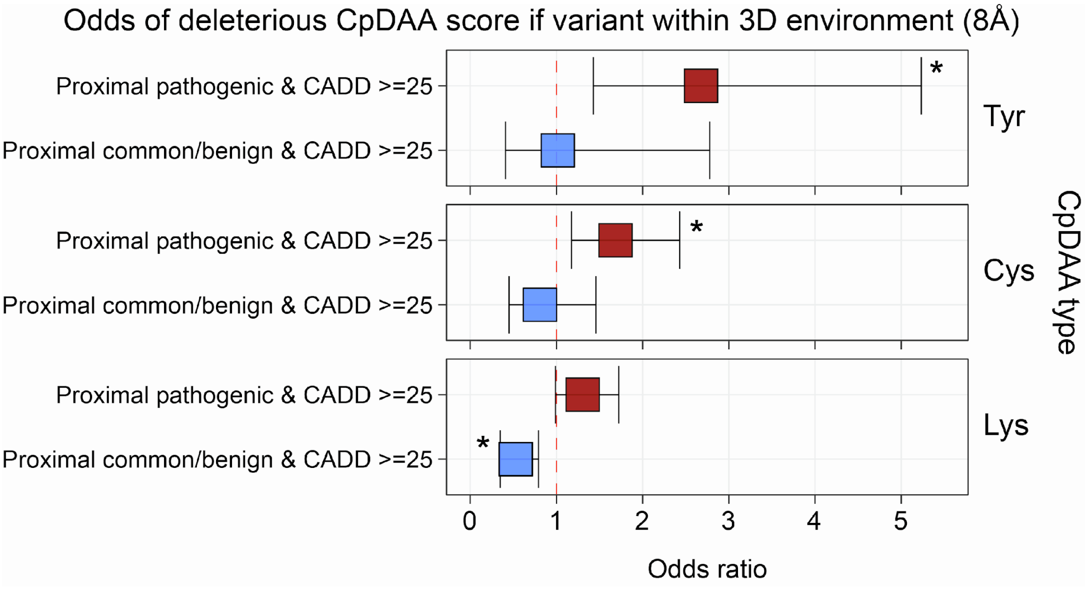

Figure S17. Odds of deleterious CADD score based on missense environments of specific CysLysTyr detected residues. CpDAA with missense alleles within the 8Å environment were compared to CpDAA with no local missense alleles in the 3D environment. Analysis based on 419 OMIM&CpD proteins.

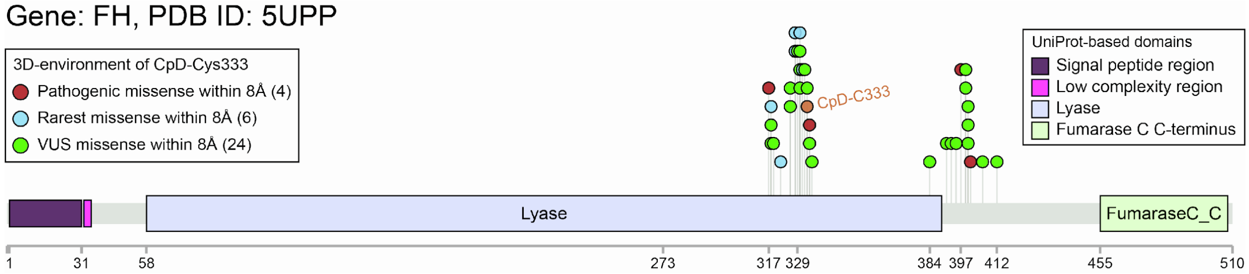

Figure S18. Missense in the 8Å environment of FH cysteine 333 shown in 1D sequence space. Figure depicting the Fumarate Hydratase gene and specific protein domains and the various pathogenic missense mutations in the 3D environment of CpD Cys333. The key observation is that the 3D space brings together regions that are separated in 1D space by ~ 30 amino acids.

Supplemental Methods

Data Sources

Data source	URL	Version
UniProtKB	https://www.uniprot.org/downloads	August 2021
HGNC	https://www.genenames.org/download/custom/	September 2020
Ensembl	https://useast.ensembl.org/info/website/archives/assembly.html	v92
gnomAD constraint	https://gnomad.broadinstitute.org/downloads#v2-constraint	2.1.1
FDA approved drug targets	http://www.proteinatlas.org	20.1; accessed May 14, 2021
OMIM	https://www.omim.org/downloads	June 24, 2021
gnomAD variants	https://gnomad.broadinstitute.org/downloads#v2-variants	2.1.1
ClinVar	https://www.ncbi.nlm.nih.gov/clinvar/	June 10, 2021
dbNSFP	https://sites.google.com/site/jpopgen/dbNSFP	4.2a

Software	URL	Version
Python	https://www.python.org/	3.7.4
R	https://www.r-project.org/	3.6.2
Tidyverse	https://doi.org/10.21105/joss.01686	1.3.0
Pandas	https://pandas.pydata.org/	0.25.1
Numpy	https://numpy.org/	1.17.2
SciPy	https://www.scipy.org/	1.3.1

Adobe Illustrator	Adobe, Inc	

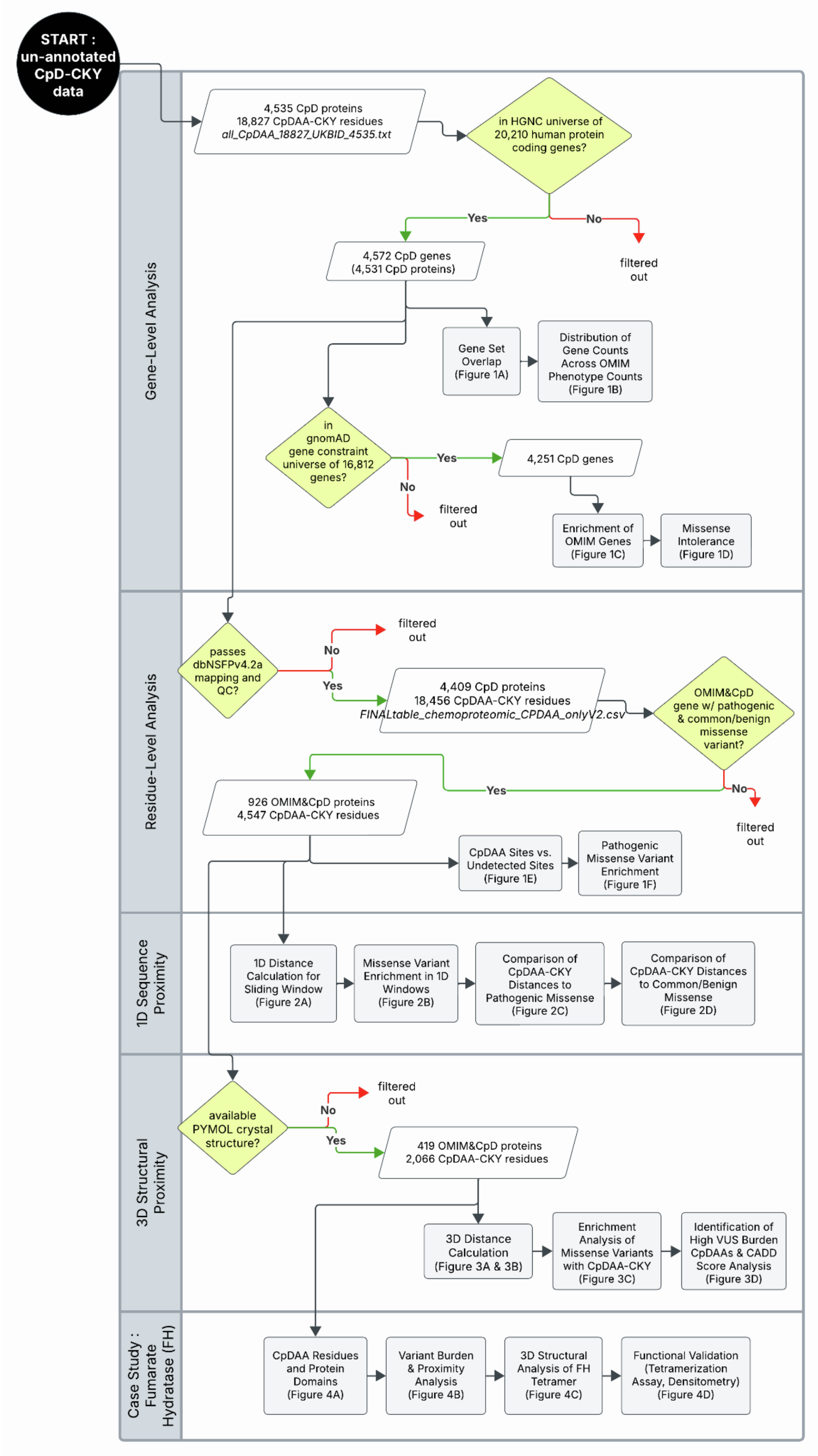

START : un-annotated CpD-CKY data
Gene-Level Analysis
4,535 CpD proteins
18,827 CpDAA-CKY residues
all_CpDAA_18827_UKBID_4535.txt
in HGNC universe of 20,210 human protein coding genes?
Yes
No
filtered out
4,572 CpD genes
(4,531 CpD proteins)
Gene Set Overlap (Figure 1A)
Distribution of Gene Counts Across OMIM Phenotype Counts (Figure 1B)
in gnomAD gene constraint universe of 16,812 genes?
Yes
No
filtered out
4,251 CpD genes
Enrichment of OMIM Genes (Figure 1C)
Missense Intolerance (Figure 1D)
Residue-Level Analysis
passes dbNSFPv4.2a mapping and QC?
No
Yes
filtered out
4,409 CpD proteins
18,456 CpDAA-CKY residues
FINALtable_chemoproteomic_CPDAA_onlyV2.csv
OMIM&CpD gene w/ pathogenic & common/benign missense variant?
Yes
No
filtered out
926 OMIM&CpD proteins
4,547 CpDAA-CKY residues
CpDAA Sites vs. Undetected Sites (Figure 1E)
Pathogenic Missense Variant Enrichment (Figure 1F)
1D Sequence Proximity
1D Distance Calculation for Sliding Window (Figure 2A)
Missense Variant Enrichment in 1D Windows (Figure 2B)
Comparison of CpDAA-CKY Distances to Pathogenic Missense (Figure 2C)
Comparison of CpDAA-CKY Distances to Common/Benign Missense (Figure 2D)
3D Structural Proximity
available PYMOL crystal structure?
No
Yes
filtered out
419 OMIM&CpD proteins
2,066 CpDAA-CKY residues
3D Distance Calculation (Figure 3A & 3B)
Enrichment Analysis of Missense Variants with CpDAA-CKY (Figure 3C)
Identification of High VUS Burden CpDAAs & CADD Score Analysis (Figure 3D)
Case Study : Fumarate Hydratase (FH)
CpDAA Residues and Protein Domains (Figure 4A)
Variant Burden & Proximity Analysis (Figure 4B)
3D Structural Analysis of FH Tetramer (Figure 4C)
Functional Validation (Tetramerization Assay, Densitometry) (Figure 4D)

Activity diagram of main analyses and results. The diagram summarizes our analyses, data sources, and key annotations. A black circle marks the Start Node. Parallelograms represent data, with unique counts annotated within each shape. Grey squares indicate main analysis steps. Yellow diamonds denote Decision Nodes, with outcomes in red (no) and green (yes). The five major analysis sections are organized in swim lanes, with analysis groups labeled on the right against grey backgrounds. The all_CpDAA_18827_UKBID_4535.txt file contains merged, unannotated CpDAA-CysLysTyr datasets that served as our starting point. The FINALtable_chemoproteomic_CPDAA_onlyV2.csv file contains 4,409 CpD proteins with 2,644,250 unique amino acid positions, including 18,456 CpDAA-CysLysTyr positions. We successfully mapped each residue to dbNSFPv4.2a pathogenicity score annotations, accepting a minimal data loss of 2.78% (n=126) CpD proteins and 1.97% (n=371) CpDAA-CysLysTyr residues compared to the merged table (all_CpDAA_18827_UKBID_4535.txt). Using dbNSFPv4.2a, we mapped CysLysTyr residues to CADD scores—a key metric for missense pathogenicity prediction. During UniProt-to-dbNSFPv4.2a mapping, some CpD proteins were filtered out due to our rigorous sequence verification process. Our requirement for complete CADD scores for all possible non-synonymous SNVs per codon also led to some protein exclusions. Despite the small data loss, this careful approach ensured high-quality missense-level annotations across all remaining CpD protein positions.
